# Porous Structure Enhances the Longitudinal Piezoelectric Coefficient and Electromechanical Coupling Coefficient of Lead‐Free (Ba_0.85_Ca_0.15_)(Zr_0.1_Ti_0.9_)O_3_

**DOI:** 10.1002/advs.202406255

**Published:** 2024-08-29

**Authors:** Zihe Li, James Roscow, Hamideh Khanbareh, Philip R. Davies, Guifang Han, Jingyu Qin, Geoff Haswell, Daniel Wolverson, Chris Bowen

**Affiliations:** ^1^ Centre for Integrated Materials Processes & Structures Department of Mechanical Engineering University of Bath Claverton Down Bath BA27AY UK; ^2^ Cardiff Catalysis Institute School of Chemistry Cardiff University Park Place Cardiff CF10 3AT UK; ^3^ Key Laboratory for Liquid‐Solid Structural Evolution and Processing of Materials (Ministry of Education) School of Materials Science and Engineering Shandong University Jinan 250061 China; ^4^ EMD Ltd. The Old Manse 29 St Mary St Ilkeston Derbyshire DE78AB UK; ^5^ Centre for Photonics and Photonic Materials and Centre for Nanoscience and Nanotechnology Department of Physics University of Bath Claverton Down Bath BA27AY UK

**Keywords:** defect engineering, electromechanical coupling coefficient, internal bias field, piezoelectric coefficient, porous ferroelectric ceramics

## Abstract

The introduction of porosity into ferroelectric ceramics can decrease the effective permittivity, thereby enhancing the open circuit voltage and electrical energy generated by the direct piezoelectric effect. However, the decrease in the longitudinal piezoelectric coefficient (*d*_33_) with increasing porosity levels currently limiting the range of pore fractions that can be employed. By introducing aligned lamellar pores into (Ba_0.85_Ca_0.15_)(Zr_0.1_Ti_0.9_)O_3_, this paper demonstrates an unusual 22–41% enhancement in the *d*_33_ compared to its dense counterpart. This unique combination of high *d*_33_ and a low permittivity leads to a significantly improved voltage coefficient (*g*_33_), energy harvesting figure of merit (*FoM*_33_) and electromechanical coupling coefficient ($k_{33}^{2}$). The underlying mechanism for the improved properties is demonstrated to be a synergy between the low defect concentration and high internal polarizing field within the porous lamellar structure. This work provides insights into the design of porous ferroelectrics for applications related to sensors, energy harvesters, and actuators.

## Graphical abstract

Introducing aligned pores into lead‐free (Ba_0.85_Ca_0.15_)(Zr_0.1_Ti_0.9_)O_3_ ceramics enhances the *d*_33_ piezoelectric coefficient by 22–41% compared to its dense counterpart, significantly improving the voltage coefficient (*g*_33_), energy harvesting figure of merit (*FoM*_33_) and electromechanical coupling coefficient ($k_{33}^{2}$). The findings provide insights into the role of porosity on the properties of ferroelectric ceramics for applications related to sensing, harvesting, and actuating devices.

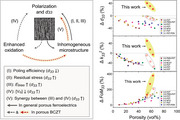

## 1 Introduction

Piezoelectricity enables a direct conversion between mechanical energy and electrical energy via the direct and converse piezoelectric effects, respectively. Among the range of available piezoelectric materials, polycrystalline ferroelectric ceramics are widely used in commercial piezoelectric devices since they exhibit a high piezoelectric response and tailored polarization orientation. Since polycrystalline ferroelectric ceramics can also be manufactured at a relatively low cost, particularly when compared to single crystal ferroelectrics, they have been successfully applied to a range of applications which include actuation,^[^ ^1^ ^]^ sensing,^[^ ^2^ ^]^ ultrasonics,^[^ ^3^ ^]^ energy harvesting^[^ ^4^ ^]^ and catalysis.^[^ ^5^ ^]^

In the context of ferroelectric materials, the concept of porosity engineering is a technique whereby pores are intentionally introduced into the structure to increase specific figures of merit, usually for applications related to energy harvesting and sensing,^[^ ^6^ ^]^ including SONAR.^[^ ^7^ ^]^ In the last few decades, this technique has been shown to be an effective approach for enhancing the open circuit voltage and electrical energy generated from an applied mechanical load via the direct piezoelectric effect. The widely accepted mechanism for this enhancement is that the introduction of porosity reduces the permittivity of the material whilst also maintaining a relatively high longitudinal piezoelectric charge coefficient, *d*_33_, which is a measure of the charge per unit force. As a result, the introduction of porosity leads to an increase in the piezoelectric voltage coefficient (*g*_33_), which is a measure of the electric field per unit stress and is of interest for sensing applications, whereby

(1) $$g_{33}=\frac{d_{33}}{\varepsilon_{33}^{\sigma }}\;$$

where $\varepsilon_{33}^{\sigma }$ is the permittivity, the superscript σ indicates a condition of constant stress, and the 33 subscript indicates the directions of the electric field and response, respectively. A low permittivity also increases the piezoelectric energy harvesting figure of merit, *FoM*_33_, whereby

(2) $$FoM_{33}=\frac{d_{33}^{2}}{\varepsilon_{33}^{\sigma }}\;=d_{33}\;\times g_{33}$$

This figure of merit has been widely used to describe the electrical energy density generated by a material for applied stress,^[^ ^8^, ^9^, ^10^ ^]^ including porous ferroelectrics. In Ref. [11] Topolov et al. fabricated a porous lead zirconate titanate (PZT) with a 3‐0 structure, where the 3 and 0 represent the dimensional connectivity of the ceramic phase and air phase in the porous ferroelectric, respectively. For a porosity fraction of ≈25 vol%, the $\varepsilon_{33}^{\sigma }$ of the porous PZT decreased to ≈20% of the dense material, while the *d*_33_ remained relatively high at ≈65% of the dense material. As a result, the *FoM*_33_ of the material doubled. When the porosity fraction was further increased to ≈60 vol%, the $\varepsilon_{33}^{\sigma }$ of the material reduced to ≈8% of the dense material. However, the introduction of a high level of porosity led to a significant decrease in *d*_33_ to ≈27% of the dense material, so that the *FoM*_33_ was ≈10% lower than that of the dense material. Similar results have also been experimentally observed in 3–0 and 3–3 porous ferroelectric ceramics,^[^ ^12^, ^13^, ^14^ ^]^ whereby the *d*_33_ decreases significantly when the porosity fraction exceeds ≈50 vol%. This decrease in the *d*_33_ at high pore fractions therefore limits the maximum potential improvement in the *FoM*_33_ via the introduction of porosity to ≈100% compared to the pore‐free material.

For applications that use the mechanical strain generated by the converse piezoelectric effect, such as actuators,^[^ ^15^ ^]^ motors,^[^ ^16^ ^]^ and ultrasonic devices,^[^ ^3^ ^]^ porosity engineering has been explored to a lesser extent. One of the main reasons for this is that the introduction of porosity reduces the electromechanical coupling coefficient, $k_{33}^{2}$, which is an important characteristic for the selection of commercial actuator materials, in particular for those operating at resonance,^[^ ^17^ ^]^ whereby,

(3) $$k_{33}^{2}=\frac{d_{33}^{2}}{\varepsilon_{33}^{\sigma }s_{33}^{E}}$$

where $s_{33}^{E}$ is the elastic compliance of the material under a constant electric field. The $k_{33}^{2}$ parameter describes the efficiency of the conversion of an electrical energy input into a mechanical energy output, and vice versa. In the case of porous ferroelectric ceramics, since the introduction of porosity reduces the Young's modulus (*Y*, ≈$1/s_{33}^{E}$) and $\varepsilon_{33}^{\sigma }$ by similar magnitudes,^[^ ^18^, ^19^ ^]^ the change in the $k_{33}^{2}$ is primarily dependent on the *d*_33_, which decreases with the introduction of porosity. Moreover, since $k_{33}^{2}$ is proportional to $d_{33}^{2}$, the impact of a decrease in *d*_33_ with increasing levels of porosity is amplified. As an example, on introducing 32 vol% of porosity into a 3–0 magnesium niobate‐lead zirconate titanate (PMN‐PZT) material, the $k_{33}^{2}$ was reduced to 50% of the dense ceramic.^[^ ^19^ ^]^ A more significant decrease in the $k_{33}^{2}$ was reported by Li et al.,^[^ ^14^ ^]^ where a 3–0 PZT material with a porosity fraction of ≈65 vol% exhibited a $k_{33}^{2}$ that was one third that of the dense material.

The gradual decrease in the *d*_33_ with the introduction of porosity remains a core barrier in the further development and application of porous ferroelectric ceramics. This issue limits any further improvement in the *g*_33_ and *FoM*_33_ and, to date, there is always a decrease in the $k_{33}^{2}$ as porosity is introduced into a ferroelectric microstructure. The decrease in *d*_33_ with increasing pore fraction has been primarily attributed to a mechanism whereby the low permittivity pores act to decrease the magnitude of the electric field within the high permittivity ferroelectric material during the poling process, which reduces the poling efficiency.^[^ ^20^ ^]^ Using finite element analysis, Roscow et al.^[^ ^21^ ^]^ and Zhang et al.^[^ ^20^ ^]^ found that, when the ceramic and pore phases are connected in series, the electric field applied during poling concentrates in the pore space due to the low permittivity of the air phase; this leads to a lower average local electric field in the ceramic phase compared to that of a dense structure. Moreover, in the poling field direction, the ferroelectric regions that are located directly above and below the pore spaces were found to be subject to a significantly lower electric field compared to other regions of the ceramic microstructure. These two issues reduce the poling efficiency of porous ferroelectric ceramics, resulting in a reduced *d*_33_, particularly at high volume fractions of porosity.

To solve the challenges associated with the electric field distribution during poling and resulting polarization, researchers have developed ferroelectric ceramics with aligned porous structures. These have included both 2–2^[^ ^22^, ^23^ ^]^ and 3–1^[^ ^24^, ^25^ ^]^ structures, whereby highly anisometric pores have been aligned along the poling field direction using a directional forming process, such as freeze casting. Such structures effectively avoid the serial connection of ceramic and air phases in the direction of the poling field and lead to improved poling efficiency. As a result, the decrease in *d*_33_ at high porosity fractions was reduced compared to the first‐generation porous ferroelectric ceramics that contained randomly distributed pores with a 3–0 or 3–3 structure. In Ref. [24] a 3–1 porous PZT was fabricated with a porosity fraction of 69 vol% and the *d*_33_ remained relatively high at 88% of the dense material of the same composition. As a result, the *FoM*_33_ was improved by ≈200–250% compared to the dense material. A higher pore volume fraction was studied by Lee et al.,^[^ ^25^ ^]^ where 82 vol% of porosity was introduced into a 3–1 porous lead zinc niobate‐lead zirconate titanate (PZN‐PZT), and the *d*_33_ was maintained as ≈65% of the dense material, increasing the *FoM*_33_ by almost a factor of three. Nevertheless, a reduction in the *d*_33_ due to the introduction of porosity was still observed. Compared to the first‐generation of porous ferroelectric ceramics, the second generation of porous ceramics, which are based on an aligned porous structure, exhibit of slower decrease in *d*_33_ with increasing pore volume fraction. This results in a maximum improvement in the *FoM*_33_ of ≈250% compared to the pore free material. The proposed explanations for the reduced *d*_33_^[^ ^20^ ^]^ were that i) the orientation of the 2–2 and 3–1 porous structures was not perfectly aligned along the poling direction, ii) the presence of serial connected pores located within the individual ceramic channels, which reduced the poling efficiency, and iii) ceramic bridges within pore channels remained unpoled and led to a degree of mechanical clamping of the poled ceramic regions, thereby reducing the *d*_33_.

To eliminate the negative effect of porosity on the *d*_33_, a third‐generation of porous ferroelectric ceramics are now being developed. In addition to engineering the local electric field and polarization of the porous structure, researchers have explored novel mechanisms to maintain a high *d*_33_ within a porous ferroelectric. A mechanism that has been successfully used to enhance the *d*_33_ was based on a “declamping” effect,^[^ ^26^ ^]^ which reduced the residual stress of the material, thereby enhancing the degree of domain switching when an electric field is applied.

In this work, an aligned porous structure is intentionally introduced into (Ba_0.85_Ca_0.15_)(Zr_0.1_Ti_0.9_)O_3_ (BCZT) lead‐free ferroelectric ceramics, a material whose piezoelectric and ferroelectric properties are sensitive to the degree of oxygen access during sintering.^[^ ^27^, ^28^, ^29^ ^]^ Two novel mechanisms were identified by which the aligned porous structure *enhanced* the piezoelectric *d*_33_ coefficient compared to the dense material. First, the presence of open pores increased the degree of oxidation, thereby reducing the concentration of oxygen vacancies. The lower concentration of oxygen vacancies acted to soften the ferroelectric material, thereby improving domain mobility and enhancing the polarization. Second, the inhomogeneous arrangement of dipoles within the porous structure generated an internal polarizing field (*E_bias_*) along the direction of polarization. Consequently, the enhanced polarization due to the low oxygen vacancy concentration generated a larger *E_bias_*, further enhancing the polarization of the material. These findings provide a unique approach to controlling the electromechanical properties of porous ferroelectrics through a synergistic combination of defect engineering and porosity engineering.

As a result of the two novel enhancement mechanisms identified in the porous BCZT fabricated in this work, an increase in the *d*_33_ was observed due to the introduction of porosity. As a result, a breakthrough was achieved in the piezoelectric properties of the porous BCZT, whereby the introduction of porosity i) increased the *d*_33_ by 31%, and *g*_33_ by 183%, compared to the dense material, ii) increased the $k_{33}^{2}$ by 16%, rather than a reduction which is commonly observed, and iii) improved the harvesting figure of merit, *FoM*_33_, by ≈400% compared to the dense material. This work therefore provides a new and effective approach to enhancing the *d*_33_, *g*_33_, $k_{33}^{2}$ and *FoM*_33_ of ferroelectric ceramics. Moreover, the insights and the synergetic effects of porosity on the properties of porous ferroelectric ceramics can be applied to a range of applications such as sensors (*g*_33_), energy harvesters (*FoM*_33_), and actuators (*d*_33_, $k_{33}^{2}$).

## 2 Results and Discussion

### 2.1 Design of Microstructure and Degree of Access to Air During Sintering

**Figure 1:**
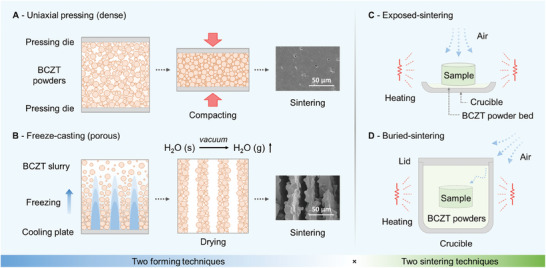
Fabrication of the BCZT ceramics. Two forming techniques were employed: A) uniaxial‐pressing for dense ceramics and B) freeze‐casting for porous ceramics, and two sintering conditions were employed: C) exposed‐sintering and D) buried‐sintering.

BCZT powders were fabricated via a solid‐reaction route, in which BaCO_3_, CaCO_3_, ZrO_2_, and TiO_2_ were used as the raw materials. The powders were mixed according to the stoichiometric ratio and calcinated at 1250 °C. X‐ray diffraction (XRD) analysis and scanning electron micrographs (SEM) of the powders are shown in Figure S1 (Supporting Information). During the fabrication of the BCZT ceramics, the microstructure was controlled by the forming techniques shown in **Figure** **1**. Conventional uniaxial pressing and sintering (Figure 1A) were used to fabricate the dense ceramics, where BCZT powders with 0.5 wt.% organic binders (polyvinyl alcohol (PVA)) were compacted in a die using a uniaxial pressure to form a BCZT ceramic green body. After sintering, a dense ceramic was obtained, see Figure S2 (Supporting Information), which exhibited a relative density of 97% and a low porosity of 3 vol.%, as shown in Figure S3 (Supporting Information). A high volume fraction of aligned porosity (up to ≈60 vol.%) was introduced into the BCZT ceramics by freeze‐casting (Figure 1B). On directionally freezing the BCZT slurry, ice crystals grew directionally along the temperature gradient, which pushed the BCZT powder particles between the aligned ice crystal walls. The ice was then removed by freeze‐drying at low pressure, leaving behind a highly aligned lamellar porous structure. This porous structure was retained after sintering, see Figure S4 (Supporting Information), and the porous BCZT ceramics had a relative density of 42% and a high porosity of 58 vol.%.

For both the BCZT ceramics with dense and porous microstructures, the degree of access to air during high‐temperature sintering was controlled by changing the sintering conditions. This approach was taken to examine the influence of greater access to air and level of oxidation during sintering, in particular for the porous materials. By using a conventional “*exposed‐sintering*” regime (Figure 1C), the dense and porous samples were fully exposed to an air atmosphere during sintering. In contrast, during a “*buried‐sintering*” regime (Figure 1D), the dense and porous samples were buried in a slightly compacted BCZT powder during sintering to reduce access to air. As shown in Figure S3 (Supporting Information), the nature of the sintering techniques had little influence on the final density of the BCZT ceramics. With the combination of the two forming/processing techniques and the two sintering regimes, four groups of BCZT ceramics were fabricated which are termed i) *dense‐exposed*, ii) *dense‐buried*, iii) *porous‐exposed*, and iv) *porous‐buried* in the remainder of this paper.

**Figure 2:**
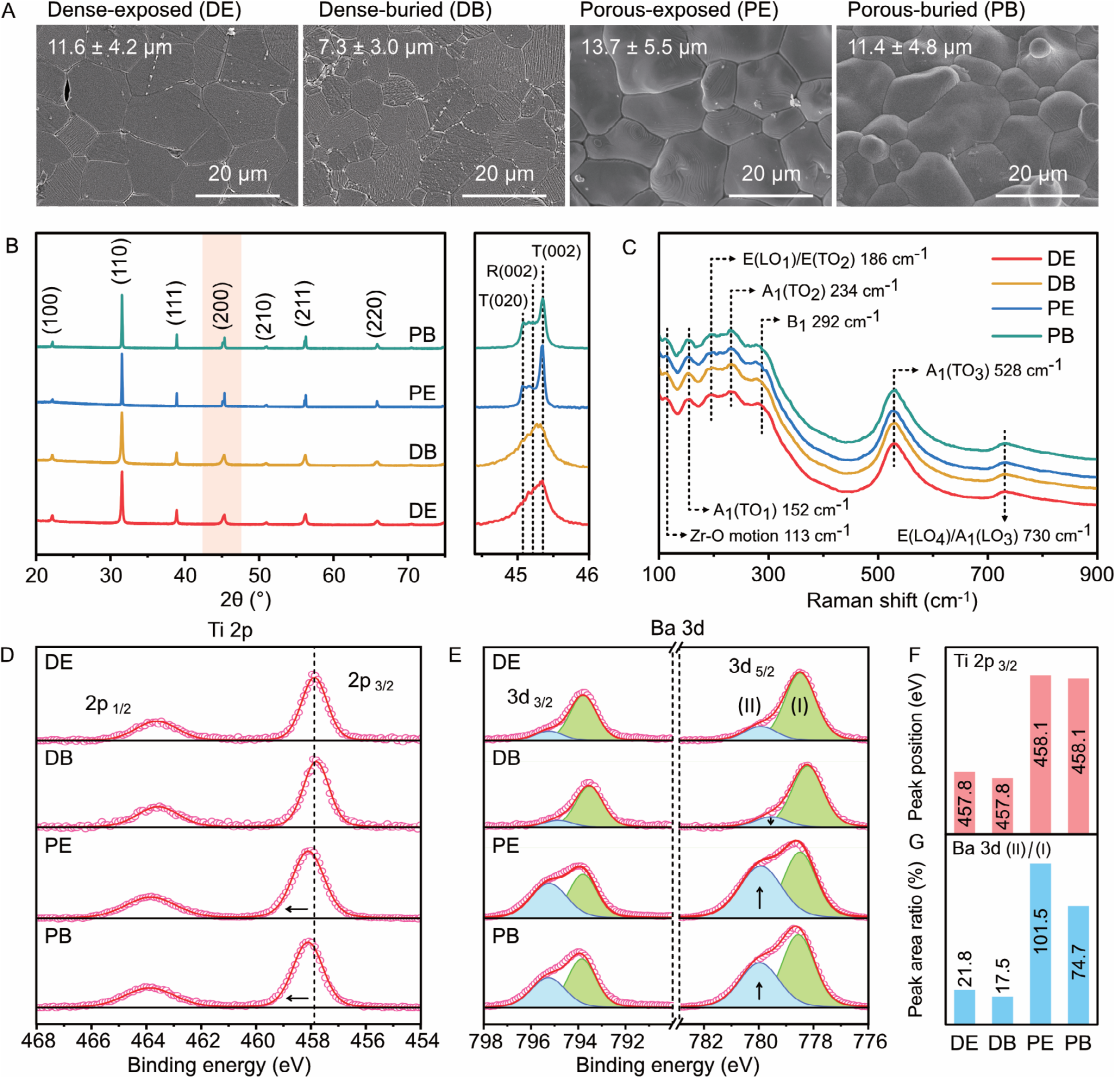
Grain microstructures and phase composition, and oxidation state of the BCZT ceramics. A) Scanning electron microscopy (SEM) of grain microstructures. B) X‐ray diffraction analysis (XRD). C) Raman spectra. High‐resolution X‐ray photoelectron spectroscopy (XPS) of D) Ti 2p and E) Ba 3d, and analysis on their F) peak position and G) peak area.

The grain structures of the BCZT ceramics are shown in **Figure** **2**A, and the grain size distribution is shown in Figure S6 (Supporting Information). All microstructures were observed to be well‐sintered, and the average grain sizes ranged from 7.3 to 13.7 µm with uniform distribution. Interestingly, the dense‐buried (7.3 ± 3.0 µm) and the porous‐buried (11.4 ± 4.8 µm) materials have a lower grain size than dense‐exposed (11.6 ± 4.2 µm) and porous‐exposed (13.7 ± 5.5 µm), where an increase in grain size is reported to enhance the polarization and *d*_33_ of BCZT.^[^ ^30^ ^]^ According to Ref. [30] the *d*_33_ of BCZT ceramics increases by ≈6 pC N^−1^ as the grain size increases per 1 µm. The crystalline structure and phase composition of the fabricated ceramics were characterized by XRD, as shown in Figure 2B. All four samples exhibited similar peak positions and intensity, which matched with the XRD of BCZT ceramics reported elsewhere.^[^ ^30^, ^31^ ^]^ Several studies have reported on the mixed‐phase composition of BCZT ceramics, including orthorhombic + tetragonal,^[^ ^32^, ^33^ ^]^ rhombohedral + tetragonal,^[^ ^30^, ^34^, ^35^ ^]^ and rhombohedral + orthorhombic + tetragonal^[^ ^27^ ^]^ phase mixtures. The variation in the exact phase composition may be due to factors such as the raw materials, the BCZT powder fabrication route, and sintering techniques and conditions. Therefore, care should be taken in the determination of the mixed‐phase composition of BCZT ceramics. Herein, first, to determine the number of the crystalline phases in the BCZT ceramics, differential scanning calorimetry (DSC) was conducted, see Figure S7 (Supporting Information). The endothermic peak at ≈30 °C indicates a mixture of two phases at room temperature. A second endothermic peak was observed at ≈100 °C, which corresponds to the Curie point (*T_C_*).

Second, to determine the crystal structures of the two phases, the XRD at (200) and (111) lattice planes were examined. In the magnified XRD of the (200) lattice planes at a 2θ of ≈45°, peak splitting was observed with a relatively weak peak component at ≈45.1° and a stronger peak at ≈45.8°.^[^ ^36^, ^37^, ^38^ ^]^ This is a characteristic of the tetragonal phase due to the larger lattice distance of the (002) planes than that of the (020) planes.^[^ ^39^ ^]^ The non‐tetragonal phase in the BCZT ceramics was determined by the asymmetric XRD peak of the (111) lattice plane at 2θ of ≈39°, as shown in Figure S8 (Supporting Information). This asymmetric peak is due to the peak splitting of the (111) plane with a larger lattice parameter and the (1$\bar{1}$1) plane with a smaller lattice parameter, which is a characteristic of the rhombohedral phase.^[^ ^39^ ^]^ Therefore, the BCZT ceramics fabricated in this work exhibit a mixed‐phase composition of rhombohedral + tetragonal, and the approximate XRD peak positions were noted in the magnified XRD patterns in Figure 2B and Figure S8 (Supporting Information) by tetragonal (*T*) and rhombohedral (*R*) phases, respectively. The content of the phase composition and lattice parameters were further determined by Rietveld refinement,^[^ ^40^ ^]^ as shown in Figure S9 and Table S1 (Supporting Information), respectively. Interestingly, the introduction of porosity and the use of an exposed‐sintering regime increased the fraction of the tetragonal phase present in the material. For BaTiO_3_‐based ceramics, the crystal phase transformation into a tetragonal structure is accompanied by internal stress due to the unequal dimensional variations along the *c*‐axis and the *a*‐ and *b*‐axis,^[^ ^41^ ^]^ which inhibits the formation of the tetragonal phase.^[^ ^41^ ^]^ In Ref. [26] it was shown that the introduction of porosity helped to release the internal stress within the material compared to the dense structure, which promoted the formation of the tetragonal phase. The exposed‐sintering regime also reduced the concentration of oxygen vacancies within the material (to be discussed later) and as a point defect, oxygen vacancies can lead to lattice distortion^[^ ^42^ ^]^ and increased internal stress within the material.^[^ ^43^ ^]^ Therefore, a lower oxygen vacancy concentration due to an exposed sintering regime or the presence of porosity can reduce the internal stress within the material, thereby enhancing the formation of the tetragonal phase.

The phase mixture was verified with Raman spectroscopy as shown in Figure 2C, in which the four groups of BCZT showed similar spectra. Clear peaks were observed at 234, 528, and 730 cm^−1^, related to the vibration modes of A_1_(TO_2_), A_1_(TO_3_), and E(LO_4_)/A_1_(LO_3_), respectively, matching with the Raman spectra of BCZT ceramics reported elsewhere.^[^ ^44^ ^]^ The simultaneous presence of the vibration modes of Zr‐O motion in the lattice at 113 cm^−1^, A_1_(TO_1_) at 152 cm^−1^ and E(LO_1_)/E(TO_2_) at 186 cm^−1^ in Figure 2C are characteristic features of the rhombohedral phase BCZT,^[^ ^45^ ^]^ and the broad shoulder, rather than a sharp peak, at 280 cm^−1^ is a signature of the tetragonal phase BCZT.^[^ ^44^ ^]^ A detailed peak assignment of the Raman data is shown in Figure S10 (Supporting Information).

To examine the nature of the chemical bonding within each of the four materials, we employed X‐ray photoelectron spectroscopy (XPS) which has been widely used to investigate the oxidation state of materials.^[^ ^46^, ^47^, ^48^ ^]^ It was used here to investigate the influence of the forming method (dense uniaxial pressing and porous freeze casting) and sintering regime (exposed and buried) on the state of oxidation of the BCZT ceramics. The XPS data was calibrated using the peak position of the adventitious carbon peak,^[^ ^49^ ^]^ as shown in Figure S5 (Supporting Information). In the high‐resolution XPS spectra of Ti 2p (Figure 2D), a peak shift toward a higher binding energy of ≈0.3 eV (Figure 2F) was observed for both the porous‐exposed and porous‐buried BCZT compared to the dense‐exposed and dense‐buried BCZT, indicating a decrease in electron density around the Ti atom.^[^ ^48^ ^]^ Due to the resolution of the XPS peak position obtained, a detailed understanding of the influence of the impact of exposed‐sintering and buried‐sintering was difficult to obtain from the XPS spectra of Ti 2p. In the XPS spectra of Ba 3d, see Figure 2E, the curve was fitted via two components, namely Ba 3d (II) with a high binding energy and Ba 3d (I) with a low binding energy, and the peak area ratio of Ba 3d(II) to Ba 3d(I) varied between samples. A detailed peak area analysis is provided in Figure 2G, in which the use of a buried‐sintering regime decreased the peak area ratio of Ba 3d(II) to Ba 3d(I) for both the dense and porous BCZT. This indicates that the use of a buried‐sintering regime, which limits access to air, inhibits the degree of oxidation of the BCZT, and increases the oxygen vacancy concentration. As an example, an increase in the peak area ratio of Ba 3d(II) to Ba 3d(I) has been reported to be related to a low oxygen vacancy concentration in barium‐based ferroelectric ceramics on sintering at high oxygen partial pressures^[^ ^50^ ^]^ and donor doping.^[^ ^51^ ^]^

In summary, structural characterization demonstrated that the four groups of BCZT ceramics were well‐sintered and contained a mixture of tetragonal and rhombohedral phases. The introduction of porosity and the use of an exposed‐sintering regime increased the fraction of the tetragonal phase present in the material, as indicated by the more significant XRD peak splitting of the (200) lattice plane (Figure 2B) and the phase content analysis based on the Rietveld refinement of the XRD data (Figure S9, Supporting Information. Compared to the dense structure, the creation of an aligned porous structure led to an increased degree of oxidation of the BCZT after sintering. The use of a buried‐sintering regime inhibited the oxidation of the BCZT material, regardless of whether the material was dense or porous. Therefore, on sintering in the same atmosphere, the introduction of open porosity into the BCZT material improved access to air during sintering, which increased the degree of oxidation and reduced the oxygen vacancy concentration. This, in turn, has an impact on the ferroelectric and piezoelectric properties, which are now discussed.

### 2.2 Ferroelectric and Piezoelectric Properties

**Figure 3:**
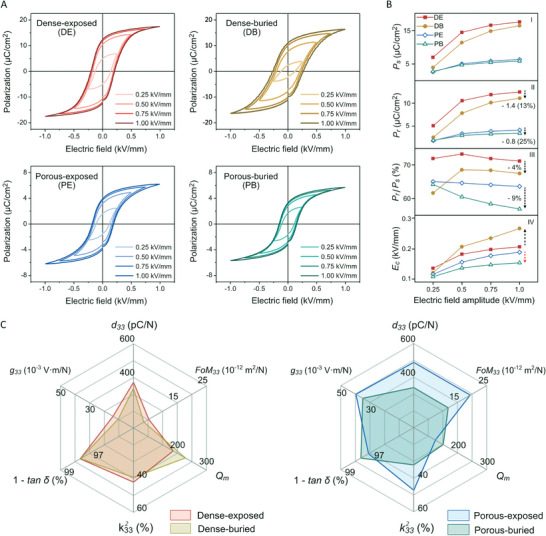
Ferroelectric and electromechanical properties of the BCZT ceramics. A) Polarization‐electric field (*P*‐*E*) hysteresis loops of the BCZT ceramics were measured at 1 Hz. B) Electric field amplitude (*E*_0_) dependence of I) saturated polarization (*P_s_*), II) remnant polarization (*P_r_*), III) squareness of *P*‐*E* loop (*P_r_*/*P_s_*) and IV) coercive field (*E_c_*). C) Longitudinal piezoelectric charge coefficient (*d*_33_), longitudinal voltage coefficient ($g_{33}=d_{33}\;/\varepsilon_{33}^{\sigma }$) at 100 Hz, longitudinal piezoelectric energy harvesting figure of merit (*FoM*_33_ = *d*_33_ *g*_33_) at 100 Hz, the electromechanical coupling coefficient of a thickness mode ($k_{33}^{2}$), dielectric loss (tan δ) at 10 kHz and mechanical quality factor (*Q_m_*).

**Figure** **3**A shows the polarization‐electric field (*P*‐*E*) hysteresis loops of the four types of BCZT ceramics investigated. The amplitude of the applied electric field (*E*_0_) ranged from 0.25 to 1.00 kV mm^−1^ with field increments of 0.25 kV mm^−1^. The saturated polarization (*P_s_*), remnant polarization (*P_r_*), squareness of the hysteresis loop (*P_r_*/*P_s_*)^[^ ^52^ ^]^ (or known as hysteresis rectangularity factor^[^ ^53^ ^]^) and coercive field (*E_c_*) are provided in Table S2 (Supporting Information), and their dependence on *E*_0_ is shown in Figure 3B. On increasing the applied electric field, both the *P_s_* and *P_r_* of the four BCZT ceramics increased, see Figure 2B‐I,II. The introduction of porosity reduced both the *P_s_* and *P_r_*. At the maximum field of *E*_0_ = 1.00 kV mm^−1^, the *P_s_* and *P_r_* of the porous BCZT were ≈35% and ≈30% of the dense BCZT, respectively. The use of a buried‐sintering regime, which restricted access to air and increased the concentration of oxygen vacancies, resulted in a decrease in the *P_s_* and *P_r_* for both the dense and porous BCZT, compared to the exposed‐sintering regime. The *P_r_*/*P_s_* ratio is shown in Figure 2B‐III, which describes the degree of polarization that is retained on removal of the applied external field; for a single crystal the *P_r_*/*P_s_* should be close to unity.^[^ ^52^ ^]^ The porous structures yielded a lower *P_r_*/*P_s_* compared with the dense material and this may be due to a declamping effect associated with the porous structure that decreases the energy barrier for domain switching on removal of the applied field. As a result, a larger fraction of saturated polarization of the porous BCZT switched back to the unpoled state on the removal of the electric field compared to the dense material. The use of buried‐sintering, which increased the concentration of oxygen vacancies, resulted in a lower *P_r_*/*P_s_* for the porous‐buried material (*P_r_*/*P_s_* = 56%) compared to porous‐exposed material (*P_r_*/*P_s_* = 65%). This reduction was twice as large as that observed for the dense materials, whereas for dense‐buried *P_r_*/*P_s_* = 67%, while for dense‐exposed *P_r_*/*P_s_* = 71%.

The coercive field, *E_c_*, is the electric field that is necessary to reduce the polarization from *P_r_* to zero and indicates the electric field required to switch ferroelectric domains. As shown in Figure 3B–IV, the *E_c_* of the porous material decreased compared to the dense material when the hysteresis loops were fully saturated as a result of a declamping effect due to the presence of porosity. However, there was no consistent trend for the influence of the sintering condition on *E_c_*; specifically, the use of a buried‐sintering regime increased the *E_c_* of the dense BCZT but decreased the *E_c_* of the porous BCZT. The underlying reason for this contrasting behavior will be explained in detail in Section 2.4. A comparison of the ferroelectric properties of the BCZT fabricated in this work and other reports can be seen in Table S2 (Supporting Information).

The piezoelectric properties and resulting performance figures of merit of the BCZT ceramics are summarised in Figure 3C and Table S3 (Supporting Information). For both the dense and porous materials, the use of a buried‐sintering regime, which leads to a higher oxygen vacancy concentration and hardening, resulted in a lower longitudinal piezoelectric charge coefficient (*d*_33_), voltage coefficient (*g*_33_), energy harvesting figure of merit (*FoM*_33_) and electromechanical coupling coefficient ($k_{33}^{2})$ compared to an exposed‐sintering regime. The calculated voltage coefficient, *g*_33_, and harvesting figure of merit, *FoM*_33_, are based on the *d*_33_ charge coefficient and the measured relative permittivity at constant stress at 100 Hz, as summarized in Figure S11 (Supporting Information). In addition, the use of a buried‐sintering regime led to a higher mechanical quality factor (*Q_m_*) and lower dielectric loss (tan δ) in both the dense and porous BCZT; this also indicates a hardening of the material due to the increased oxygen vacancy concentration. A comparison of the piezoelectric properties of the BCZT fabricated in this work and other reports can be seen in Table S3 (Supporting Information).

A key observation from these data is that the introduction of highly aligned porosity into BCZT has been shown to *increase* the *d*_33_ coefficient by 31% compared to the dense BCZT, which has successfully overcome the conventional observation that the introduction of porosity reduces the *d*_33_. This has led to the porous material exhibiting an improved voltage coefficient (*g*_33_), energy harvesting figure of merit (*FoM*_33_), and electromechanical coupling coefficient ($k_{33}^{2}$). In addition, the use of a buried‐sintering regime (compared to an exposed‐sintering regime) resulted in a larger decrease in the *d*_33_ and *P_r_* for the porous material, compared to the dense material. To provide further insight into this phenomenon, the polarization and switching mechanisms for the four types of BCZT material and the resulting evolution of the lattice structure with applied field were investigated using synchrotron XRD, which is now described.

**Figure 4:**
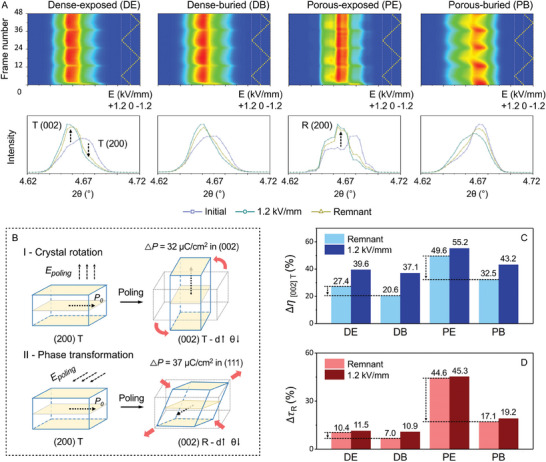
Polarization and switching mechanisms of the BCZT ceramics. A) High energy synchrotron XRD analysis of the BCZT ceramics from the (002) lattice plane with a detecting direction parallel to the applied electric field (Figure S12, Supporting Information) under in situ application of a bipolar electric field (*E*) with a peak amplitude of 1.2 kV mm^−1^ and step size of 0.2 kV/mm. B) Schematics of the two mechanisms showing the change of polarization due to the applied field: I) crystal rotation and II) phase transformation (tetragonal to rhombohedral); *P*_0_ = spontaneous polarization, Δ*P* = polarization change, *d* = distance between adjacent lattice planes. C) Change of the orientation degree of [002] in the tetragonal phase (Δη_[002]T_). D) Change of volume fraction of the rhombohedral phase (Δτ_*R*_).

**Figure** **4**A shows high energy synchrotron XRD data for the (002) lattice plane of bulk samples in transmission mode with in situ electric fields applied during measurement, which was obtained in the direction parallel to the applied electric field, i.e., with an azimuthal angle of zero, see Figure S12 (Supporting Information). A cyclic bipolar electric field (*E*) was applied to the four materials with a peak amplitude 1.2 kV mm^−1^ in increments of 0.2 kV mm^−1^, generating 48 XRD patterns for each sample. A contour plot showing the peak position of the tetragonal (002) lattice plane, defined as (002) T, as a function of the electric field is shown in the upper row of Figure 4A. The 48 XRD patterns correspond to 48 frame numbers on the *y*‐axis label, where the *x*‐axis shows the 2*θ* angle of the XRD, and the red and blue colors represent high and low peak XRD intensities, respectively. For the four types of BCZT ceramic, the contour plots exhibited periodic patterns every 12 frame numbers, as the electric field varied from 0 to ±1.2 kV mm^−1^. Within individual cycles of the applied field, the lattice structure of the BCZT begins in the unpoled state of polarization and then switches between the maximum polarization and remnant polarization states. The 2D XRD patterns for these three polarization states are shown in the lower row of Figure 4A. The XRD data were similar in the maximum and remnant polarization states, but a significant difference was observed during the initial polarization cycle.

First, on the application of the electric field, the intensity of the (002) T peak at 4.65° increased and the intensity of the (200) T peak at 4.68° decreased, as indicated by the arrows in Figure 4A. This can be attributed to the *crystal rotation* of the tetragonal phase, shown in Figure 4B‐I, where the spontaneous polarization of the lattice (*P*_0_) is aligned parallel to the applied field. Second, the intensity of the (200) rhombohedral peak at 4.67° increased as the electric field was applied; the corresponding mechanism is illustrated in Figure 4B‐II, where the electric field leads to a *phase transformation* from a tetragonal to rhombohedral phase so that both the magnitude and orientation of *P*_0_ is changed.

To determine the change in polarization (Δ*P*_0_) via these two polarization mechanisms, density functional theory (DFT) calculations were conducted. The approximate lattice structure of BCZT was obtained, as shown in Figure S13 (Supporting Information), and the calculated lattice parameters and properties are provided in Table S4 (Supporting Information). In terms of crystal rotation, the maximum Δ*P* reached 32 µC cm^−2^, which was obtained when *P*_0_ switched from [200] to [002]. The maximum Δ*P*_0_ of the phase transformation occurred when *P*_0_ switched from [−200] to [111], generating a Δ*P*_0_ of 37 µC cm^−2^. Therefore, both polarization‐switching mechanisms contribute to the overall polarization of the material. Since the above two polarization mechanisms can switch the *P*_0_ orientation of the lattice, both crystal rotation and phase transformation are classified as *extrinsic* effects which are related to domain motion in response to an applied electric field.

To further study the difference in the above two polarization mechanisms for the four types of BCZT materials, peak fitting of the synchrotron XRD data was conducted to quantitatively analyze the phase fraction and domain orientation as a function of the applied electric field, see Figure S14 (Supporting Information). To assess the *extrinsic* contribution due to crystal rotation, the percentage of ferroelectric domains orientated along the [002] direction at a specific condition, $\eta_{[002]T}^{E}$, was calculated from:^[^ ^26^, ^54^ ^]^

(4) $$\eta_{\left[002\right]T}^{E}=\;\frac{I_{\left[002\right]T}^{E}}{I_{\left[002\right]T}^{E}+2\frac{I_{\left[002\right]T}^{0}}{I_{\left[200\right]T}^{0}}\times I_{\left[200\right]T}^{E}}\times 100\%$$

where $I_{[002]T}^{E}$ and $I_{[200]T}^{E}$ were the intensity of the (002) T peak at 4.65° and the (200) T peak at 4.68° under the applied electric field, respectively. The superscript ‘0′ for $I_{[002]T}^{0}$ and $I_{[200]T}^{0}$ refers to the unpoled state of polarization. The change in η_[002]*T*_ due to domain switching due to crystal rotation, denoted as Δη_[002]*T*_, with applied electric field can then be quantified as

(5) $$\Delta \eta_{\left[002\right]T}=\left(\eta_{\left[002\right]T}^{E}-\eta_{\left[002\right]T}^{0}\right)\times 100\%$$

where the $\eta_{[002]T}^{E}$ and $\eta_{[002]T}^{0}$ are defined as the fraction of the switched domains in their poled and unpoled states. As shown in Figure 4C, the introduction of porosity led to a higher fraction of domain switching by crystal rotation, Δη_[002]*T*_, compared to the dense microstructure. On considering the impact of the sintering condition, a slight decrease in the fraction of switching was observed in restricting oxygen access and increasing the oxygen vacancy concentration in the dense‐buried BCZT (Δη_[002]*T*_ = 21% for the remnant polarization state) compared to the dense‐exposed BCZT (Δη_[002]*T*_ = 27% for the remnant polarization state). In contrast, for the porous BCZT material, restricting oxygen access using a buried‐sintering regime led to a much larger decrease in Δη_[002]*T*_ for the remnant polarization, where Δη_[002]*T*_ = 50% for the porous‐exposed BCZT and Δη_[002]*T*_ = 33% for the porous‐buried BCZT.

The change in polarization resulting from the phase transformation from the tetragonal phase into the rhombohedral phase, which is the second extrinsic domain motion mechanism, is described by the volume fraction of the rhombohedral phase as^[^ ^55^ ^]^

(6) $$\tau_{R}=\frac{I_{\left(200\right)R}}{I_{\left(200\right)R}+I_{\left(200\right)T}+I_{\left(002\right)T}}$$

where *I*_(200)*R*_ is the intensity of the (200) R peak at 4.67°. In this case, the change in the τ_(200)*R*_ with the applied electric field can be described by,

(7) $$\Delta \tau_{R}=\left(\tau_{R}^{E}-\tau_{R}^{0}\right)\times 100\%$$

where the $\tau_{R}^{E}$ and $\tau_{R}^{0}$ are defined as the volume fraction of the rhombohedral phase under poled and unpoled states. As shown in Figure 4D, both the porous‐exposed and porous‐buried BCZT exhibited a significantly higher Δτ_*R*_ compared to the dense BCZT samples, indicating a larger degree of phase transformation with the applied electric field. The use of buried‐sintering, which increased the oxygen vacancy concentration, reduced the Δτ_*R*_ compared to the exposed‐sintering, and this difference was more significant in the porous BCZT compared to the dense sample. For example, for the remnant state of polarization, the porous‐exposed material showed Δτ_*R*_ = 45 vol%, while for the porous‐buried material, it reduced to Δτ_*R*_ = 17 vol%. This reduction was nine times larger than the reduction in Δτ_*R*_ from the dense‐exposed (Δτ_*R*_ = 10 vol%) to the dense‐buried (Δτ_*R*_ = 7 vol%).

While the Δη_[002]*T*_ and Δτ_*R*_ were used to assess the extrinsic contributions due to crystal rotation (domain switching) and a phase transformation, respectively, the *intrinsic* contribution to the polarization from lattice distortion was characterized by the peak shift of the (111) lattice plane, as shown in Figure S14 (Supporting Information). The dense BCZT materials exhibited a greater tensile lattice distortion, compared to the porous BCZT materials. There was no significant influence of the sintering condition of the dense‐exposed and dense‐buried BCZT; in contrast, the buried‐sintering inhibited the tensile lattice distortion in the porous‐buried BCZT compared to the porous‐exposed BCZT. This can be correlated to the higher concentration of oxygen vacancies in the porous‐buried material, where the positively charged oxygen vacancy tends to lower the spontaneous polarization of the lattice^[^ ^56^, ^57^ ^]^ and thereby reduces the strain of the lattice to the applied electric field.

In summary, while the introduction of aligned lamellar pores reduced the intrinsic polarization, it enhanced the extrinsic polarization by a declamping effect^[^ ^26^ ^]^ that increased the level of domain switching due to crystal rotation (Δη_[002]*T*_) and a phase transformation (Δτ_*R*_).

### 2.3 Porous Structures and Defect Engineering

The differences in the electromechanical properties and switching behavior between the dense‐exposed and porous‐exposed BCZT are likely due to i) the presence of a high fraction of open porosity enhancing the oxidation of BCZT during sintering, thereby reducing the oxygen vacancy concentration, and ii) microstructural effects, such declamping and the internal electric field distribution during poling. To better understand the magnitude of the effect of oxidation on properties, its influence should be separated from any possible microstructural effects. This can be achieved by making direct comparisons between the dense‐exposed and dense‐buried BCZT, and between the porous‐exposed and porous‐buried BCZT, where the porous structure is consistent between samples, but there is a difference in the degree of oxidation; see Figure 2D–F.

We have shown that the use of buried‐sintering leads to i) a decreased level of oxidation and thereby a higher concentration of oxygen vacancies, ii) a lower polarization *P_s_* and *P_r_* (Figure 3A,B), iii) a lower extrinsic and intrinsic polarization (Figure 4; Figure S15, Supporting Information), and iv) a hardening effect that leads to a higher *Q_m_* and tan δ, and a reduction in *d*_33_ and $k_{33}^{2}$ (Figure 3C). A possible explanation for this observation is that the increased oxygen vacancy concentration of the material has an impact on the ferroelectric response. Oxygen vacancies can be introduced without the need for doping by controlling the sintering atmosphere, as reported by Wang et al.^[^ ^27^ ^]^ and Liu et al.^[^ ^58^ ^]^ According to defect engineering theory, point defects in the form of lattice oxygen vacancies tend to form at the site closest to the B‐site to minimize the electrostatic energy^[^ ^56^, ^57^ ^]^ and, thus, the spontaneous polarization. In addition, positively charged oxygen vacancies also generate defect dipoles, inhibiting polarization switching through *domain pinning*,^[^ ^58^, ^59^ ^]^ which hardens the material to increase *E_c_* and decrease *d*_33_. However, the concept of defect engineering does not fully align with two specific aspects observed in the experimental data. First, while the “hardened” dense‐buried BCZT exhibits a higher *E_c_* than the dense‐exposed BCZT, the porous‐buried BCZT has a lower *E_c_* compared to the porous‐exposed material. Second, compared to the property changes between dense‐exposed and dense‐buried BCZT, a large difference was observed between the porous‐exposed and porous‐buried BCZT, in particular, a two times larger change in *P_r_*, four times larger change in the *d*_33_, three times larger change in crystal rotation polarization (Δη_[002]*T*_), nine times larger change in the phase transformation polarization (Δτ_*R*_), and nine times greater change in the intrinsic polarization. Therefore, in addition to defect engineering, which is associated with the degree of oxidation, there appears to be an additional mechanism that is unique to the aligned porous structure that affects the *E_c_* and amplifies the impact of defect engineering and oxygen vacancy concentration. This is addressed in the following section in terms of an internal bias field that is present in the porous material.

### 2.4 Porous Structures and Internal Bias Field (*E_bias_*)

An internal bias field (*E_bias_*) can be produced in ferroelectric ceramics when the arrangement of dipoles is inhomogeneous.^[^ ^60^ ^]^ As a result, the position of the *P*‐*E* hysteresis loop is shifted, leading to an asymmetric loop. For example, for a dipole in a ferroelectric thin film, the number of surrounding dipoles in the plane parallel to the poling direction is significantly lower than that in the plane perpendicular to the poling direction. Such an inhomogeneous dipole arrangement can result in a depolarizing bias field, *E_bias_*,^[^ ^61^, ^62^ ^]^ which is undesirable for the application of ferroelectric memory devices^[^ ^63^, ^64^ ^]^ and capacitors.^[^ ^65^ ^]^

**Figure 5:**
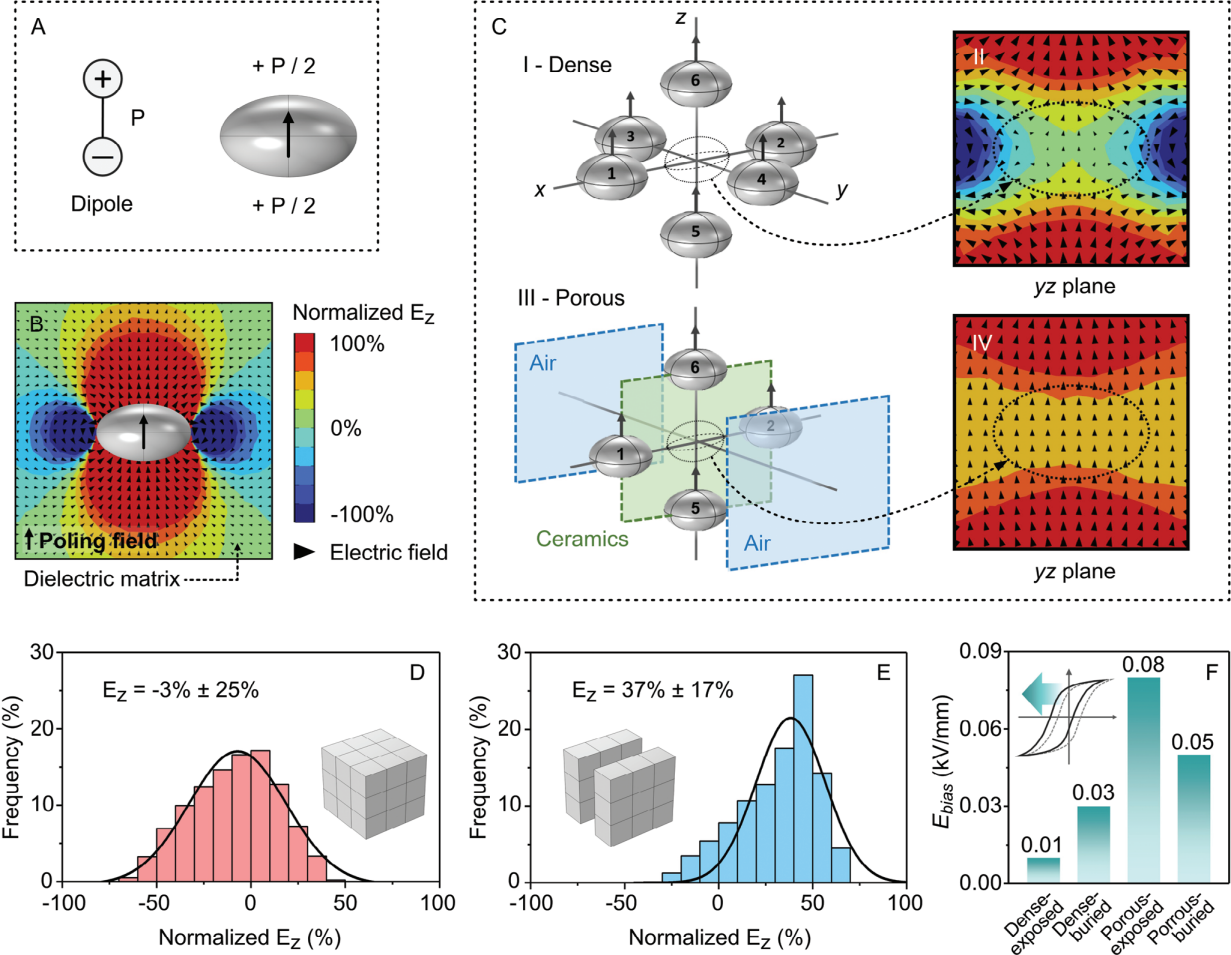
Simulation and experimental characterization of the internal bias field produced by the porous structure. A) Finite element analysis of a dipole with a polarization of *P* and B) electric field generated around the dipole. C) Finite element analysis of the influence of the microstructure on the dipole arrangement and therefore, the generated electric field: in (I, II) a 2–2 porous structure and III, IV) a dense structure. Distribution of the internal bias field in the poling direction (*E_z_*) of poled ferroelectrics with D) dense and E) 2–2 porous structures, which are normalized to the peak *E_z_* in (B). F) Experimentally measured internal bias field (*E_bias_*) of the poled BCZT ceramics from *P*‐*E* loops of poled samples (Figure S16, Supporting Information.

To examine the presence of an *E_bias_* within a porous ferroelectric, finite element analysis was conducted to provide a qualitative understanding of the impact of porosity on polarization state. In the simulation shown in **Figure** **5**A, a dielectric ellipse with an opposing charge density (+ *P*/2 and − *P*/2) at the upper and lower surfaces of the elliptical volume represents a dipole with a polarization *P*. The relative permittivity (dielectric constant) of the dielectric phases, i.e., both the dielectric ellipse and the dielectric matrix, was set at a value similar to dense BCZT, namely 3000. The electric field generated by the dipole in the surrounding dielectric matrix was then simulated, as in Figure 5B. The electric field along the polarization direction (*E_z_*) at the top and bottom surfaces of the ellipse was positive (red) and in the same direction as the dipole, which can enhance the polarization in the high‐field (red) regions in the matrix, see Figure 4B. It can be seen that *E_z_* at the left‐ and right‐hand sides of the dipole are negative (blue), which can inhibit the polarization in the blue negative‐field regions in the matrix. The influence of the microstructure on the dipole arrangement is shown in Figure 5C. In a poled dense microstructure (Figure 5C‐I), the positions around the central dipole are fully occupied by adjacent ferroelectric material/dipoles, which are numbered 1–6. Their polarizations (denoted by the up arrows) are in the same orientation since the material is poled. As a result, the *E_z_* generated by dipoles adjacent to the central dipole region contains a mixture of the positive *E_z_* located at the top and bottom surfaces (near positions 5 and 6) and the negative *E_z_* located at the left and right surfaces (near positions 1, 2, 3, and 4), as shown in Figure 5C‐II. However, for an aligned porous structure with a 2–2 connectivity, representative of those formed by freeze casting, the dipoles in positions 3 and 4 are replaced by pores/air and dipoles with a much lower dielectric constant of unity, see Figure 5C‐III. As a result, their negative (blue) contribution to the local field, *E_z_*, and the resultant negative influence on the central dipole region is much reduced, as seen in Figure 5C‐IV.

Based on these simulations, the distribution of *E_z_* in ferroelectrics with dense and porous structures, which are poled by applying an electric field, can be shown in Figure 5D,E, respectively, where the “normalized *E_z_*” is normalized with respect to the maximum *E_z_* in Figure 5B. For the dense microstructure, *E_z_* is concentrated at *E_z_* = 0%, indicating that there is a negligible bias field, *E_bias_* since there is a mix of positive (red) and negative (blue) fields in Figure 5C‐II due to the homogenous distribution of dipoles. In contrast, the *E_z_* in the porous microstructure was approximately 37% of the peak field, indicating that the presence of highly aligned pores led to an *E_bias_* in the same direction as the poling direction since no regions of negative (blue) electric fields regions are observed in Figure 5C‐IV due to the loss of dipoles in positions 3 and 4.

To experimentally determine the presence of an *E_bias_* after poling, *P*‐*E* hysteresis loops of poled BCZT samples were measured, as shown in Figure S16 (Supporting Information), and the resulting *E_bias_* was evaluated from a shift in the *P‐E* loop and compared, see Figure 5F. In agreement with the simulations, a relatively high bias field of *E_bias_* = 0.08 kV mm^−1^ and *E_bias_* = 0.05 kV mm^−1^ were observed in the poled porous‐exposed and porous‐buried BCZT ceramics, reaching 42% and 33% of their coercive field (*E_c_*), respectively. The lower *E_bias_* of the porous‐buried BCZT is due to the decreased polarization of the material due to the presence of oxygen vacancies that harden the material and hinder domain switching. In contrast, the *E_bias_* of both the dense‐exposed and dense‐buried BCZT were much smaller (0.01 and 0.03 kV mm^−1^, respectively), which is due to the homogeneous distribution of dipoles, as shown in Figure 5C,D. The slightly higher *E_bias_* of the dense‐buried BCZT, compared to dense‐exposed BCZT, indicates a higher concentration of oxygen vacancies,^[^ ^59^ ^]^ which is consistent with the XPS data (Figure 2D–G).

After validating the formation of an *E_bias_* arising due to the porous structure both experimentally and with modeling, its impact on properties is now discussed. For the aligned porous structure, since the *E_bias_* is in the same direction as the polarization of the material, it acts to enhance the overall polarization of the material. In addition to enhancing the polarization, the presence of the *E_bias_* can affect the deploying process during reversal of the applied field, and the resulting coercive field. In this case, after poling the material, the polarization of the material is reduced to zero by applying an electric field in the reverse direction. To overcome the *E_bias_*, which is established during the poling process, a larger applied electric field is required compared to a material with zero *E_bias_*. As a result, the presence of an *E_bias_* leads to an increase in the effective *E_c_* of the material. This mechanism explains the unexpected increase in *E_c_* of the porous‐exposed BCZT compared to the porous‐buried BCZT (Figure 3B‐IV), despite the softening effects that result from the lower oxygen vacancy concentration in the porous‐exposed material.

### 2.5 Porosity Dependency and Influencing Mechanisms

**Figure 6:**
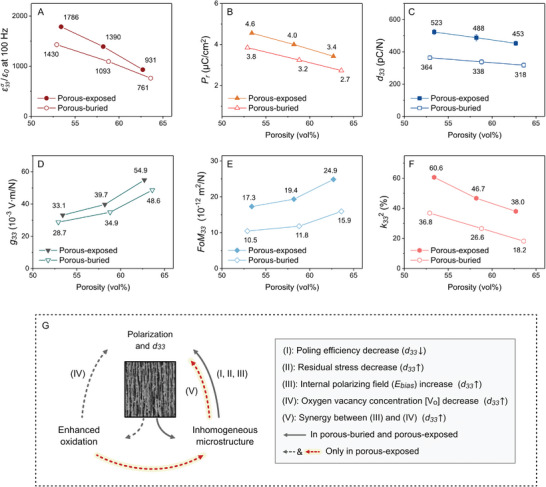
Porosity dependency of the porous BCZT of A) relatively permittivity ($\varepsilon_{33}^{\sigma }/\varepsilon_{0}$), B) remnant polarization (*P_r_*) measured at a poling field magnitude of 1 kV mm^−1^, C) longitudinal piezoelectric charge coefficient (*d*_33_), D) longitudinal voltage output constant (*g*_33_), E) longitudinal piezoelectric energy harvesting figure of merit (*FoM*_33_) and F) electromechanical coupling coefficient of a thickness mode ($k_{33}^{2}$). G) Influencing mechanisms of aligned porous structure on the polarization and *d*_33_ of porous‐exposed (PB) and porous‐buried (PB) BCZT ceramics.

In addition to control of the oxygen vacancy concentration and the internal polarizing field that results from the porous structure, other mechanisms can also influence the properties of a porous material, such as a reduced poling efficiency,^[^ ^20^ ^]^ or a decreased residual stress.^[^ ^26^ ^]^ To gain a comprehensive understanding of these impacts, the dependency of a range of material properties with porosity volume fraction was investigated. As the porosity fraction increased from 53 to 64 vol%, both the relative permittivity ($\varepsilon_{33}^{\sigma }/\varepsilon_{0}$) and remnant polarization (*P*_r_) of the porous‐exposed and porous‐buried BCZT decreased, as shown in **Figure** **6**A,B, respectively. This reduction is simply due to a lower fraction of the ferroelectric phase, where the *P*_r_ was measured from the *P*‐*E* loop with an electric field magnitude of 1 kV mm^−1^ (Figure S17, Supporting Information). A slight decrease in the longitudinal piezoelectric charge coefficient (*d*_33_) was observed with increasing pore fraction, see Figure 6C, which is due to the higher porosity disturbing the electric field distribution in the material during the poling process, thus reducing the poling efficiency.^[^ ^20^ ^]^ Consequently, the large reduction in $\varepsilon_{33}^{\sigma }/\varepsilon_{0}$ (Figure 6A) and small decrease in *d*_33_ (Figure 6C) led to a large increase in the longitudinal voltage output constant (*g*_33_) and piezoelectric energy harvesting figure of merit (*FoM*_33_) with increasing porosity, as shown in Figure 6D,E, respectively. The dependency of the thickness‐mode electromechanical coupling coefficient ($k_{33}^{2}$) on porosity is shown in Figure 6F, and as the porosity increased from 53 to 64 vol%, the $k_{33}^{2}$ was approximately halved, primarily due to the decreased *d*_33_.

The similar dependency of relative permittivity, remnant polarization (*P*_r_) and *d*_33_ with porosity fraction for both porous‐exposed and porous‐buried BCZT can be attributed to their common influencing mechanisms. Figure 6G outlines the range of mechanisms that operate in the freeze‐cast porous structure: I) a reduced poling efficiency^[^ ^20^ ^]^ due to the presence of low permittivity pores, which hinders polarization and decreases *d*_33_, II) a reduced residual stress^[^ ^26^ ^]^ due to the presence of low‐stiffness pores, which promotes polarization and *d*_33_, and III) an increased internal polarization field, which promotes polarization and *d*_33_. These mechanisms operate in both the porous‐exposed and porous‐buried BCZT.

The significant differences in the polarization (Figures 3B and 4; Figure S15, Supporting Information) and *d*_33_ (Figure 3C) between the porous‐exposed and porous‐buried BCZT materials are due to the unique characteristics of the porous‐exposed BCZT, namely due to mechanism IV), enhanced oxidation, which reduced the oxygen vacancy concentration, thereby “softening” the material. In BCZT ceramics, this enhancement in *d*_33_ has been observed in Ref. [27, 29] where BCZT sintered in an oxygen atmosphere exhibited *d*_33_ values 8–21% higher than those sintered in a nitrogen atmosphere. Notably, the *d*_33_ of the porous‐exposed BCZT in this work was 42–45% higher than that of the porous‐buried BCZT. This significant enhancement may result from a synergistic effect between mechanisms IV (oxygen concentration) and III (internal bias field), which is highlighted as mechanism (V) in Figure 6G by a dashed red arrow. First, the increased level of oxidation due to the use of an exposed‐sintering regime reduced the concentration of oxygen vacancies [*V_O_*], which softened the material and facilitated domain motion to enhance the polarization. Second, the increased polarization of the material due to the low [*V_O_*] produced a larger internal polarizing field, *E_bias_*, that further enhanced the *d*_33_, leading to a synergy between defect engineering and porosity engineering. This enhancement resulted in the *d*_33_ of the porous‐exposed BCZT being 22–41% higher than the *dense* counterpart.

**Figure 7:**
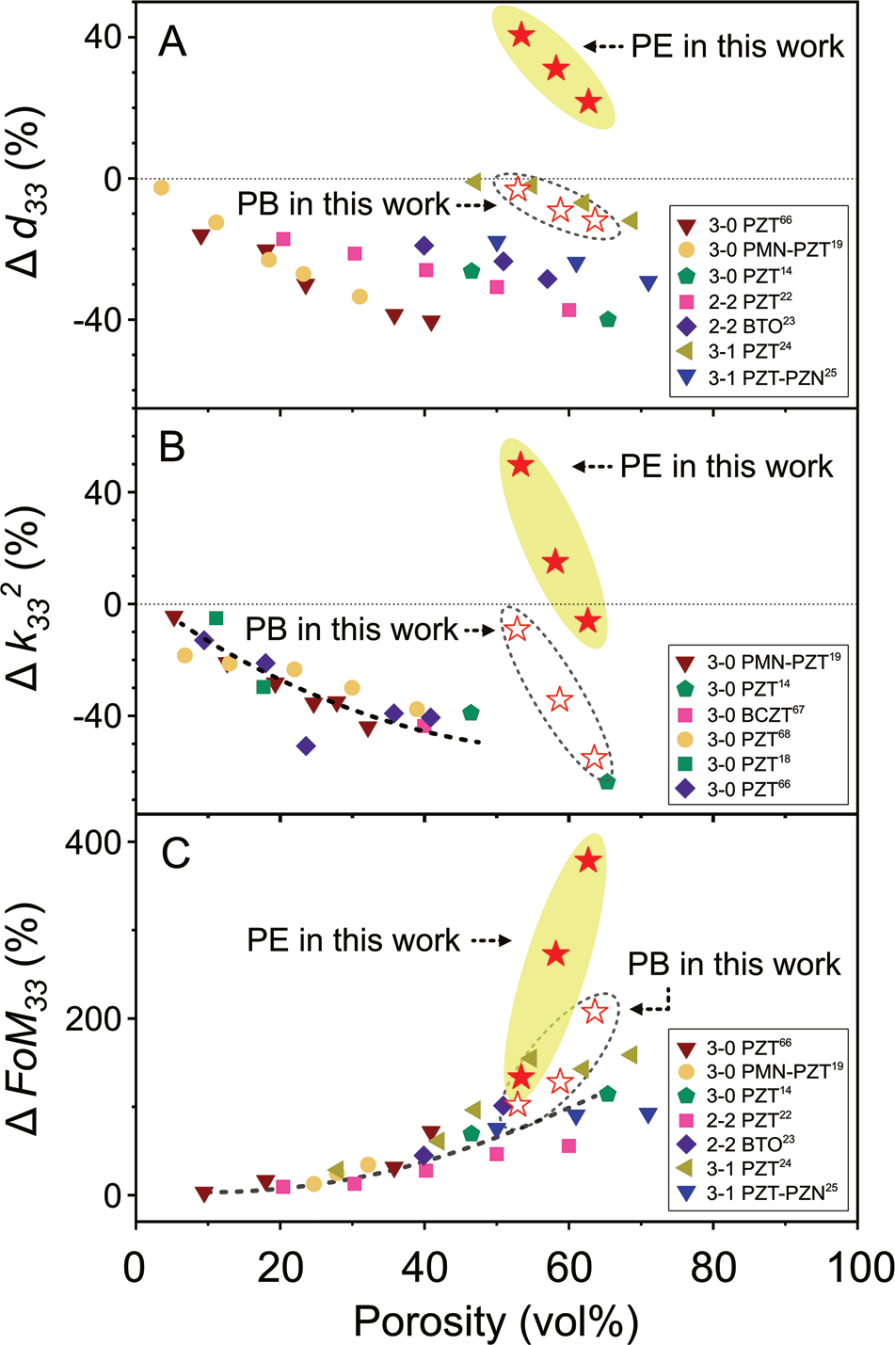
Property change of the porous‐exposed (PE) and porous‐buried (PB) BCZT ceramics compared to porous ferroelectric ceramics reported elsewhere. A) Change in longitudinal piezoelectric coefficient (Δ*d*_33_).^[^ ^14^, ^19^, ^22^, ^23^, ^24^, ^25^, ^66^ ^]^ B) Change in electromechanical coupling coefficient (Δ*k* ^2^).^[^ ^18^, ^19^, ^66^, ^67^, ^68^ ^]^ C) Change in piezoelectric energy harvesting figure of merit (Δ*FoM*_33_).^[^ ^14^, ^19^, ^22^, ^23^, ^24^, ^25^, ^66^ ^]^

The high *d*_33_ of the porous‐exposed BCZT, which was 22–41% higher than the dense material, has therefore overcome the traditional reduction in *d*_33_ that is observed when porosity is introduced into the microstructure, see **Figure** **7**A. On surveying the range of porous ferroelectric ceramics reported to date, as shown in Figure 7B, the introduction of porosity results in a decrease in the $k_{33}^{2}$, and a negative change ($\Delta k_{33}^{2}$), where the decrease in $\Delta k_{33}^{2}$ becomes larger as the fraction of porosity increases. However, due to the unique combination of a high *d*_33_ and low permittivity (Equation 3), the porous‐exposed BCZT reported here exhibited a $k_{33}^{2}$ that was up to 50% higher than that of the dense BCZT, even at a high porosity fraction of ≈53 vol%. This combination of properties breaks the conventional negative dependency of $k_{33}^{2}$ on the porosity, as indicated by the dash line in Figure 7B. Furthermore, the harvesting figure of merit, *FoM*_33_, of porous ferroelectric ceramics typically increase with increasing level of porosity, leading to a positive change (Δ*FoM*_33_). The change in the harvesting figure of merit with porosity reported to date is indicated by the dashed line in Figure 7C and at a porosity fraction of ≈60 vol%, the Δ*FoM*_33_ is typically ≈100% greater than the dense material. However, in this work, the Δ*FoM*_33_ of the porous BCZT is 377% higher than the dense materials, due to the combination of an increased *d*_33_ and reduced permittivity, which significantly exceeds previous work.

### 2.6 Energy Harvesting Performance

**Figure 8:**
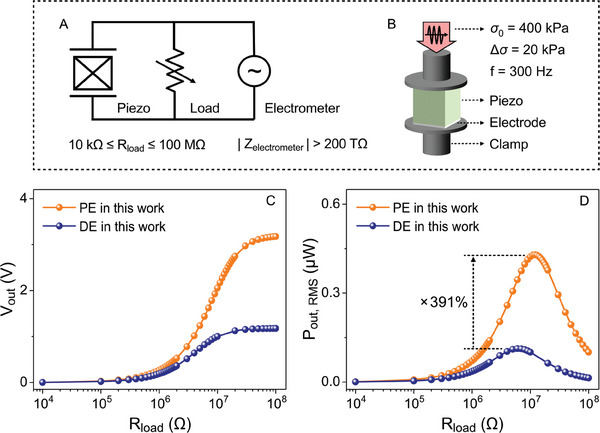
Energy harvesting performance of the dense‐exposed (DE) and porous‐exposed BCZT (PE) ceramics. A) Energy harvesting circuit diagram. B) External mechanical excitation conditions; (σ_0_ = preload stress, Δσ = fluctuating stress amplitude, *f* = stress frequency). C) Peak output voltage (*V_out_*) under load. D) Root mean squared output power (*P*_*out*,*RMS*_) under load.

To demonstrate the improvement in the energy harvesting figure of merit, *FoM*_33_, in a practical application, energy harvesting testing was conducted to compare the power output of a porous‐exposed and dense‐exposed BCZT harvester. To create the energy harvesters, BCZT ceramics were sectioned into 5 × 5 × 10 mm^3^ bars using a diamond wire saw, see Figure S18 (Supporting Information). After applying electrodes and poling the materials, the porous‐exposed and dense‐exposed BCZT samples were placed in a test circuit where the piezoelectric element was connected to a resistance decade box and an electrometer in parallel, see **Figure** **8**A. As shown in Figure 8B, a prestress (σ_0_) of 400 kPa was applied to the sample to clamp the piezoelectric material in place. A sinusoidal stress was superimposed to the prestress with an amplitude Δσ = 20 kPa and a frequency *f* = 300 Hz to represent the source of external mechanical excitation. The resulting peak piezoelectric voltage (*V_out_*) was measured using a high impedance electrometer (>200 TOhm) with the load resistance (*R_load_*) ranging from 10 kΩ to 100 MΩ, see Figure 8C. At an *R_load_* close to 100 MΩ, the *V_out_* of the both dense and porous BCZT reached a maximum, with little change in the voltage with a further increase in the load resistance, indicating that the *V_out_* was close to the open‐circuit voltage. At *R_load_* = 100 MΩ, the porous‐exposed BCZT had a *V_out_* = 3.2 V, which was 267% higher than the *V_out_* = 1.2 V of the dense‐exposed BCZT, which was similar to the improvement in the *g*_33_ of 284%. The higher *V_out_* highlights the improved sensing performance of the porous‐exposed BCZT, which is proportional to *g*_33_. With regard to energy harvesting performance, the root‐mean‐square output power (*P*_*out*, *RMS*_) of the BCZT ceramics was calculated based on the *V_out_* and *R_load_* as

(8) $$P_{out,RMS}=\frac{\;{V_{out}}^{2}}{2R_{load}}$$

The *P*_*out*,*RMS*_ power curves of both the dense and porous BCZT are shown in Figure 8D. The maximum *P*_*out*,*RMS*_ was obtained at a *R_load_* of 6 MΩ in the dense‐exposed BCZT and 12 MΩ in the porous‐exposed BCZT, i.e., the point at which the electrical impedance of the sample was matched to the circuit (ω*R_load_* *C_piezo_* =  1). The higher optimum *R_load_* for the porous material is due to its lower permittivity and device capacitance, *C_piezo_*. The maximum *P*_*out*,*RMS*_ of the porous‐exposed BCZT was 0.43 µW, which was four times higher than that of the dense‐exposed BCZT, 0.11 µW. This improvement was of a similar magnitude to that observed for the Δ*FoM*_33_ of the porous‐exposed compared to the dense‐exposed BCZT of 371%. A detailed correlation between *FoM*_33_ and power output can be seen in the energy flow model analysis^[^ ^14^ ^]^ in the supplemental information (Equations S2–S15 and Figures S19–S22, Supporting Information).

## 3 Conclusion

This paper provides the first demonstration that the formation of (Ba_0.85_Ca_0.15_)(Zr_0.1_Ti_0.9_)O_3_ (BCZT) ceramics with highly aligned pores can enhance the polarization and increase the *d*_33_ piezoelectric coefficient by 22–41% compared to its dense counterpart. This led to a significantly improved voltage coefficient (*g*_33_), energy harvesting figure of merit (*FoM*_33_) and electromechanical coupling coefficient ($k_{33}^{2}$). Using a combination of detailed characterization and modeling, the presence of porosity in the ferroelectric was shown to enhance the polarization and *d*_33_ by two main mechanisms. First, the presence of open pores improved the oxidation of the material during heat treatment, which reduced the oxygen vacancy concentration and softened the material to increase the level of polarization. Second, the inhomogeneous arrangement of dipoles within the porous structure generated an internal polarizing field (+ *E_bias_*), which is parallel to the direction of polarization. The increased polarization in the porous material, due to the low oxygen vacancy concentration, led to an increase in + *E_bias_*, which further enhanced the overall polarization of the material. As a result, the porous ferroelectric exhibited greater levels of domain switching by crystal rotation and phase transformation, compared to the dense sample.

Since porosity was shown to increase the *d*_33_ while also decreasing the permittivity, the porous BCZT exhibited a harvesting figure of merit, *FoM*_33_, which was 232–477% higher than the dense BCZT. This was consistent with the power output of the porous BCZT harvester which was four times greater than a dense BCZT harvester. This improvement also significantly broadened the upper limit of the increase in the figure of merit (Δ*FoM*_33_) when compared to existing reports on porous ferroelectric ceramics at a similar porosity level. In addition, by overcoming the often observed decrease in *d*_33_ with increasing levels of porosity, a breakthrough was achieved whereby the $\;k_{33}^{2}$ of the porous BCZT was up to 50% higher than the dense BCZT. This approach represents a novel and effective strategy to significantly enhance the piezoelectric response of ferroelectric ceramics through synergistic control of both defect engineering and porosity engineering. This work has therefore provided new insights into the role of porosity on the properties of ferroelectric ceramics for applications related to sensing, harvesting, and actuating devices.

## 4 Experimental Section

### Powder Preparation

BCZT powders were synthesized via a solid‐reaction route^[^ ^69^, ^70^ ^]^ using analytical grade (Sigma Aldrich) BaCO_3_ (purity ≥ 99%), CaCO_3_ (≥99%), ZrO_2_ (≥ 99%) and TiO_2_ (titanium (IV) oxide, rutile, 99.99% (metal basis)) as the raw materials. They were mixed according to the stoichiometric ratio and ball‐milled with yttria‐stabilized zirconia balls in ethanol for 48 h. The slurry was then dried in an oven to remove the ethanol and then ground into a powder for calcination, where the powders were heated to 1250 °C for 3 h with a heating rate of 5 °C min^−1^. The calcinated material was re‐grounded into powder and sieved through a mesh with a size of 63 µm to obtain the BCZT powders used to fabricate the dense and porous ceramics.

### Ceramic Preparation

To fabricate the dense pellets, BCZT powder was mixed with a polyvinyl alcohol (PVA) solution (10 wt.% aqueous solution) as a binder, where the weight ratio of the PVA to the BCZT powders was 0.5 wt.%. The mixture was placed in a stainless steel die and compacted using a uniaxial pressure of 80 MPa. The dense BCZT green body was dried in an oven prior to sintering. The porous BCZT was fabricated using directional freeze‐casting, where the BCZT powders were ball milled with deionized water, PVA binder, and polyacrylate dispersion agent (1 wt.% and 0.5 wt.% to the solid loading, respectively) for 48 h. The levels of solid loading (BCZT powder to water) were ≈18, 22, and 26 vol%. The suspension was poured into a polydimethylsiloxane (PDMS) mold of low thermal conductivity for freezing. Aluminum tape was applied to the base of the mold (the surface in contact with the cold plate) to hold the suspension and ensure high thermal conductivity for directional freezing. To initiate freezing, an aluminum plate was cooled to −80 °C using a Lauda Ultralow Temperature Circulator (RP290) to generate a temperature gradient from the bottom of the mold to the top of the slurry. After the slurry was frozen, the ice was removed by freeze drying under vacuum for 48 h to obtain porous BCZT green bodies. During the high‐temperature sintering process, the process of “exposed‐sintering” was conducted by placing the green BCZT bodies on a BCZT powder bed, but leaving them otherwise exposed to an air atmosphere during sintering. For the “buried‐sintering” process, the BCZT green body was buried in a mass of BCZT powder inside a corundum crucible. The crucible was shaken by hand and the powders were slightly compacted without damaging the green bodies; the crucible was then covered with a lid. All samples were subject to the same sintering conditions, where the green body was first heated to 500 °C for 2 h at a heating rate of 1 °C min^−1^ to burn out the organic binder and dispersant, prior to sintering at 1400 °C for 10 h with a heating rate of 5 °C min^−1^. The furnace was cooled to room temperature at a rate of 5 °C min^−1^.

### Characterization and Measurement

The density and porosity of the BCZT were measured by the Archimedes’ method. The crystal structure and phase composition of the BCZT powders and ceramics were determined by X‐ray diffraction measurements using CuKα radiation (XRD, BRUKER D8‐Advance). For XRD analysis of the bulk ceramic samples, they were first ground into powders. Rietveld refinement of the XRD data was conducted using GASS II.^[^ ^71^ ^]^ Raman spectra were measured by a confocal Raman microscope (LabRAM HR Evolution, Horiba Jobin Yvon) using a 455 nm laser. The microstructural features were observed by scanning electron microscopy (SEM, Hitachi SU3900). To observe the grain morphology, the polished sample was thermally etched at 1100 °C in air for 0.5 h. The grain size was measured using SEM images processed by ImageJ. The chemical environment of the BCZT ceramics was examined by X‐ray photoelectron spectroscopy (XPS, Kratos Axis SUPRA), and the phase transformation temperature of the BCZT ceramics was measured with differential scanning calorimetry (DSC, TA Instruments Q20) on bulk samples. To characterize the dielectric, piezoelectric, and ferroelectric properties of the BCZT ceramics, silver paint (RS Components, Product No. 186–3600) was coated on the material as the electrode followed by heating up to 130 °C for 0.5 h to remove the organic agent. The BCZT ceramics were poled using corona poling at 60 °C with a DC voltage of 16 kV for 15 min applied from a point source located 5 cm above the sample. The permittivity and dielectric loss of the material were measured at room temperature using an impedance analyzer (Solartron 1260 Dielectric, Hampshire). The longitudinal piezoelectric charge coefficient was measured with a Berlincourt Piezometer (PM300, Piezotest). The electromechanical coupling coefficient and mechanical quality factor were measured via an impedance analyzer (Agilent 4194A, Keysight), using the resonance‐antiresonance method on BCZT bars sectioned into dimensions of 5 × 5 × 10 mm^3^; the poling direction was along the thickness direction. The polarization‐electric field (*P*‐*E*) hysteresis loop of the BCZT ceramics was measured with a Radiant RT66B‐HVi Ferroelectric Test system. The in‐situ XRD response to the electric field of bulk BCZT ceramics was characterized by high energy synchrotron XRD experiments on Beamline I15 at Diamond Light Source‐Figure S11 (Supporting Information). XRD patterns were collected in transmission mode as a function of the applied electric field. A monochromatic photon source was used with an energy of 78.395 eV and XRD patterns were collected using a Perkin–Elmer flat panel detector. In the piezoelectric output testing, the sample was clamped using a prestress of 400 kPa and a fluctuating stress in the form of a sinusoidal superimposed waveform with an amplitude of 20 kPa at a frequency of 300 Hz. A purely resistive circuit was used to determine the piezoelectric output, in which the resistive loading was provided by a decade resistance box and the output voltage was measured using an electrometer (B2987A, Keysight) with a high electrical impedance, above 1 TΩ.

### First‐Principles Calculations

First‐principles calculations were performed in the Vienna Ab Initio Simulation Package (VASP). The approximate structure of BCZT was obtained according to Ref. [72] in which a 2 × 2 × 2 supercell barium titanate was doped with a Ca atom and a Zr atom to substitute a Ba atom and a Ti atom, respectively. The Perdew‐Burke‐Ernzerhof (PBE) pseudopotential was used with the plane‐wave energy cutoff of 420 eV. A 3 × 3 × 3 Γ‐centered k‐point mesh was used. The convergence of energy and forces acted on the ions were set at 10^−5^ eV and 0.005 eV Å^−1^, respectively.

### Finite Element Analysis

Finite element analysis was conducted in COMSOL Multiphysics. The dielectric constant along the polarization direction of the dipole and the dielectric matrix was 3000. For the air phase, the dielectric constant was assumed to be unity. A dipole with a polarization of *P* was generated in the form of an ellipse with a surface charge density with opposing polarity (± *P*/2) at the two sides. The ratio of the height to the width of the ellipse was 0.7. The thickness ratio of the ferroelectric and air layers was approximately determined according to the SEM observation, such as Figure S4 (Supporting Information).

## Conflict of Interest

The authors declare no conflict of interest.

## Supporting information

Supporting Information

## Data Availability

The data that support the findings of this study are available from the corresponding author upon reasonable request.
