# Supplementary material for: Porous Structure Enhances the Longitudinal Piezoelectric Coefficient and Electromechanical Coupling Coefficient of Lead‐Free (Ba0.85Ca0.15)(Zr0.1Ti0.9)O3
Source: Adv Sci (Weinh). 2024 Aug 29;11(40):2406255. doi: 10.1002/advs.202406255 (PMC11516054; doi:10.1002/advs.202406255)
Supplement: Supplementary file 1 — Supporting Information [file ADVS-11-2406255-s001.docx]

Supporting Information

Porous structure enhanced the longitudinal piezoelectric coefficient and electromechanical coupling coefficient of lead-free (Ba_0.85_Ca_0.15_)(Zr_0.1_Ti_0.9_)O_3_

Zihe Li*, James Roscow, Hamideh Khanbareh, Philip R. Davies, Guifang Han, Jingyu Qin, Geoff Haswell, Daniel Wolverson, Chris Bowen


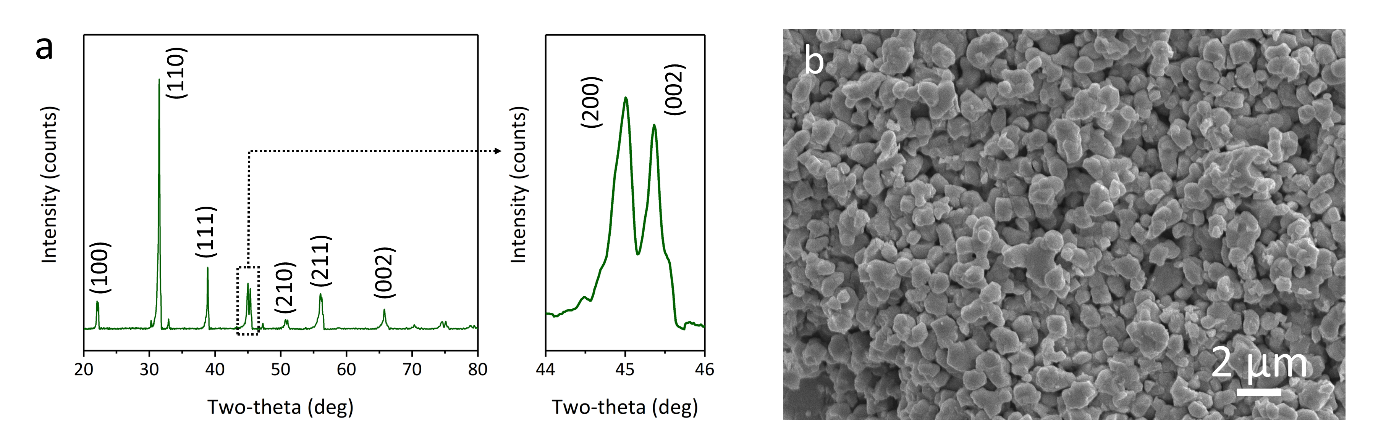


**Figure S1** Characterization of (Ba_0.85_Ca_0.15_)(Zr_0.1_Ti_0.9_)O_3_ (BCZT) powders: (a) X-ray diffraction (XRD) analysis and (b) scanning electron microscopy (SEM).


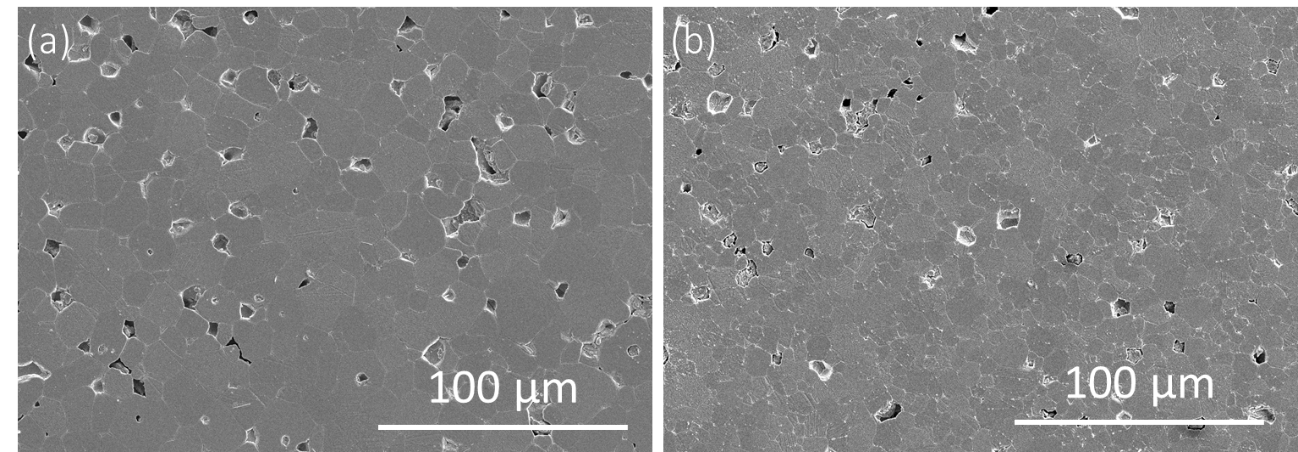


**Figure S2** Microstructures of the (a) dense-exposed BCZT and (b) dense-buried BCZT.

Since no pore forming agent was used to manufacture BCZT with a dense microstructure, the pores observed are likely to be due to material loss during the polishing process.


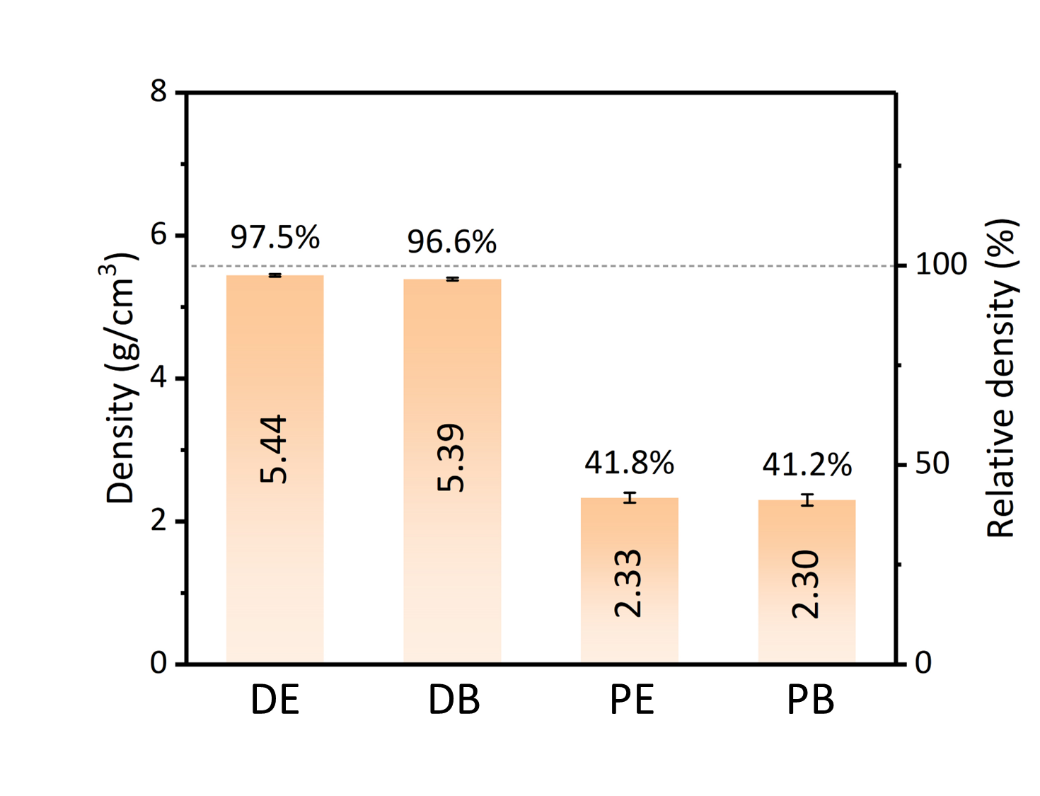


**Figure S3** Density and relative density (%) of the BCZT ceramics (DE = dense-exposed, DB = dense-buried, PE = porous-exposed, PB = porous-buried).


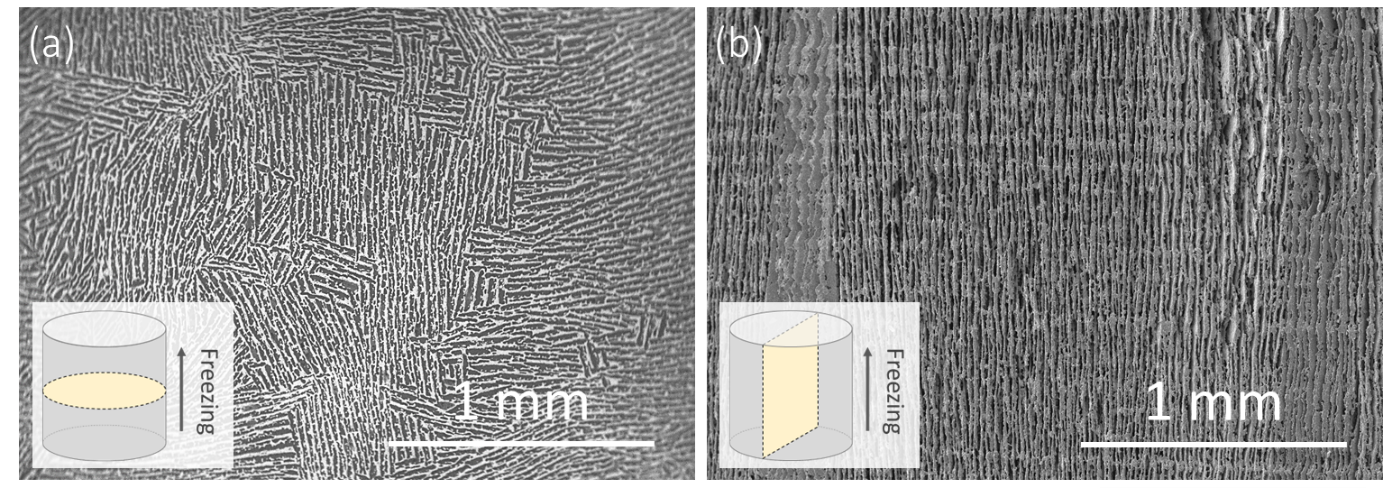


**Figure S4** Microstructure of the porous-exposed BCZT with cross sections (a) perpendicular and (b) parallel to the freezing direction.


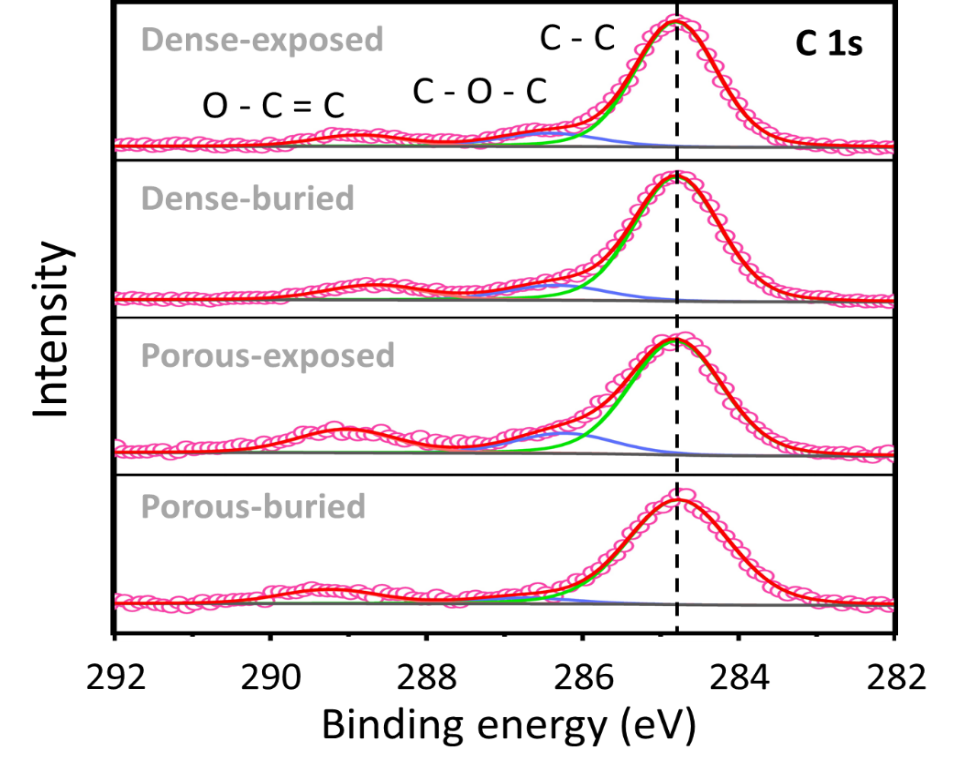


**Figure S5** X-ray photoelectron spectroscopy (XPS) of BCZT ceramics of C 1s.


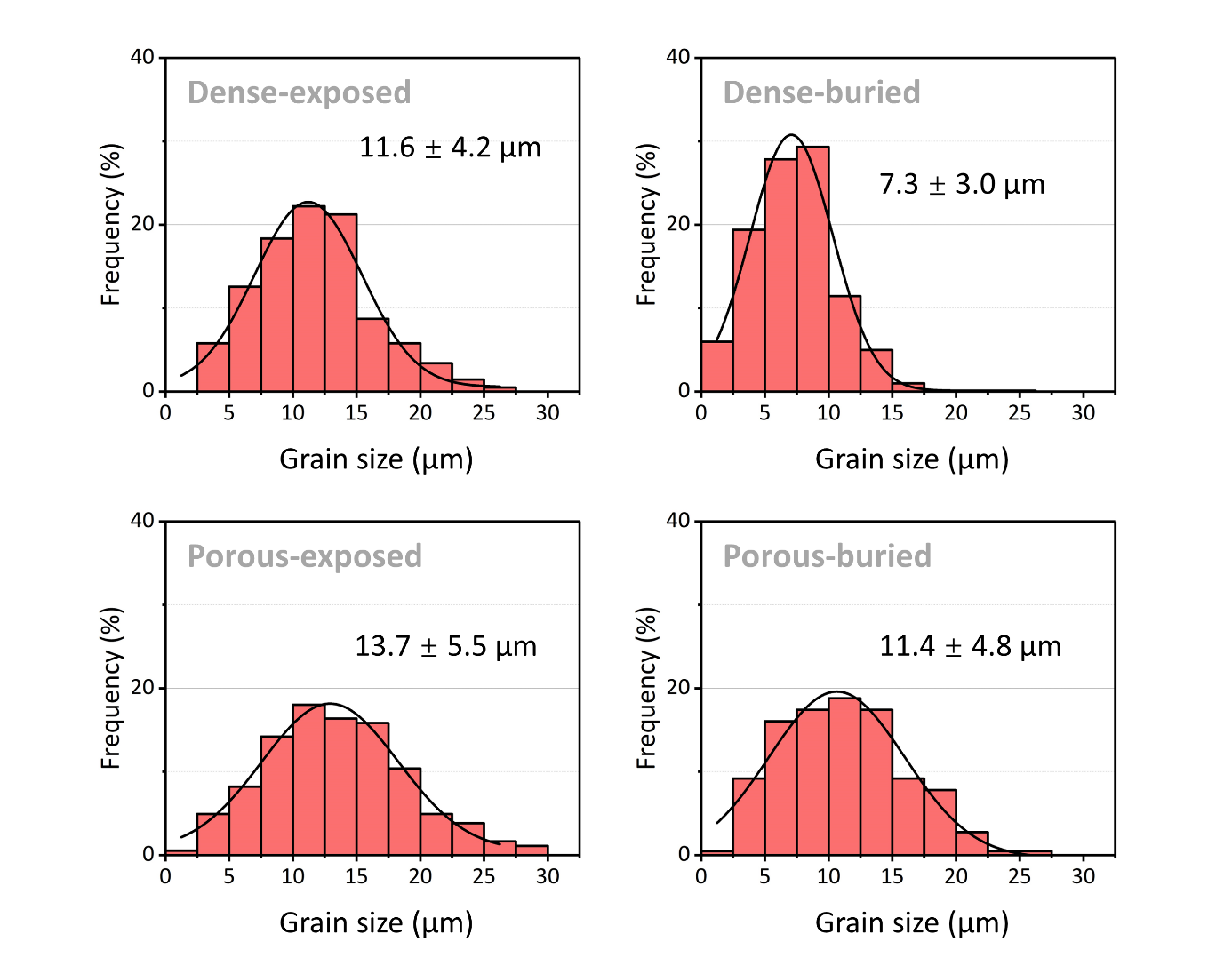


**Figure S6** Grain size distribution of BCZT ceramics.


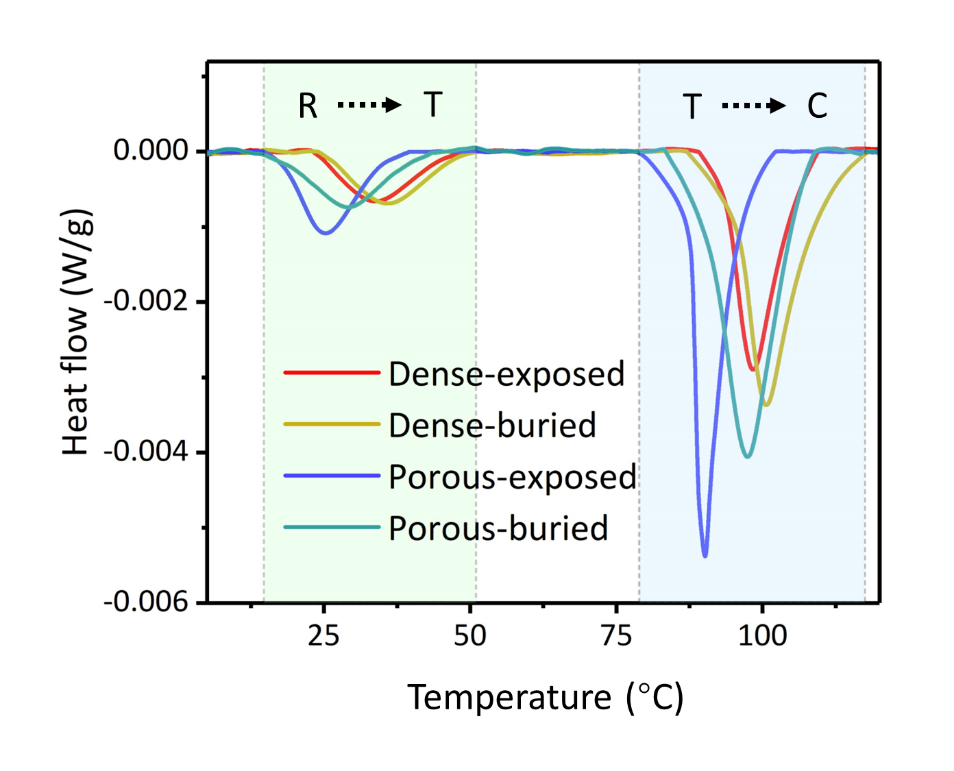


**Figure S7** Differential scanning calorimetry (DSC) of BCZT ceramics.


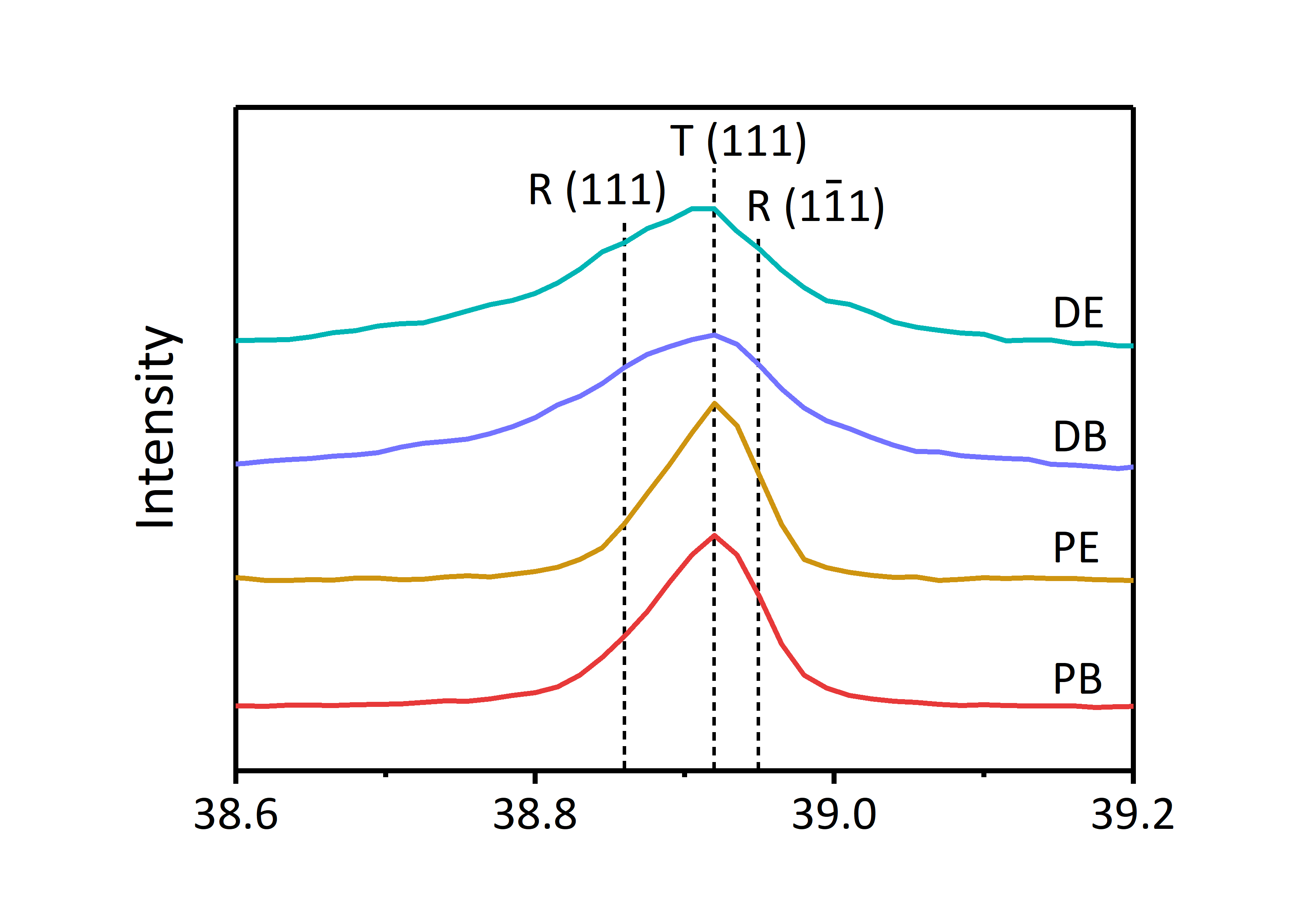


**Figure S8** Magnified X-ray diffraction (XRD) of BCZT ceramics at the (111) lattice plane (DE = dense-exposed, DB = dense-buried, PE = porous-exposed, PB = porous-buried, R = rhombohedral, T = tetragonal).

**
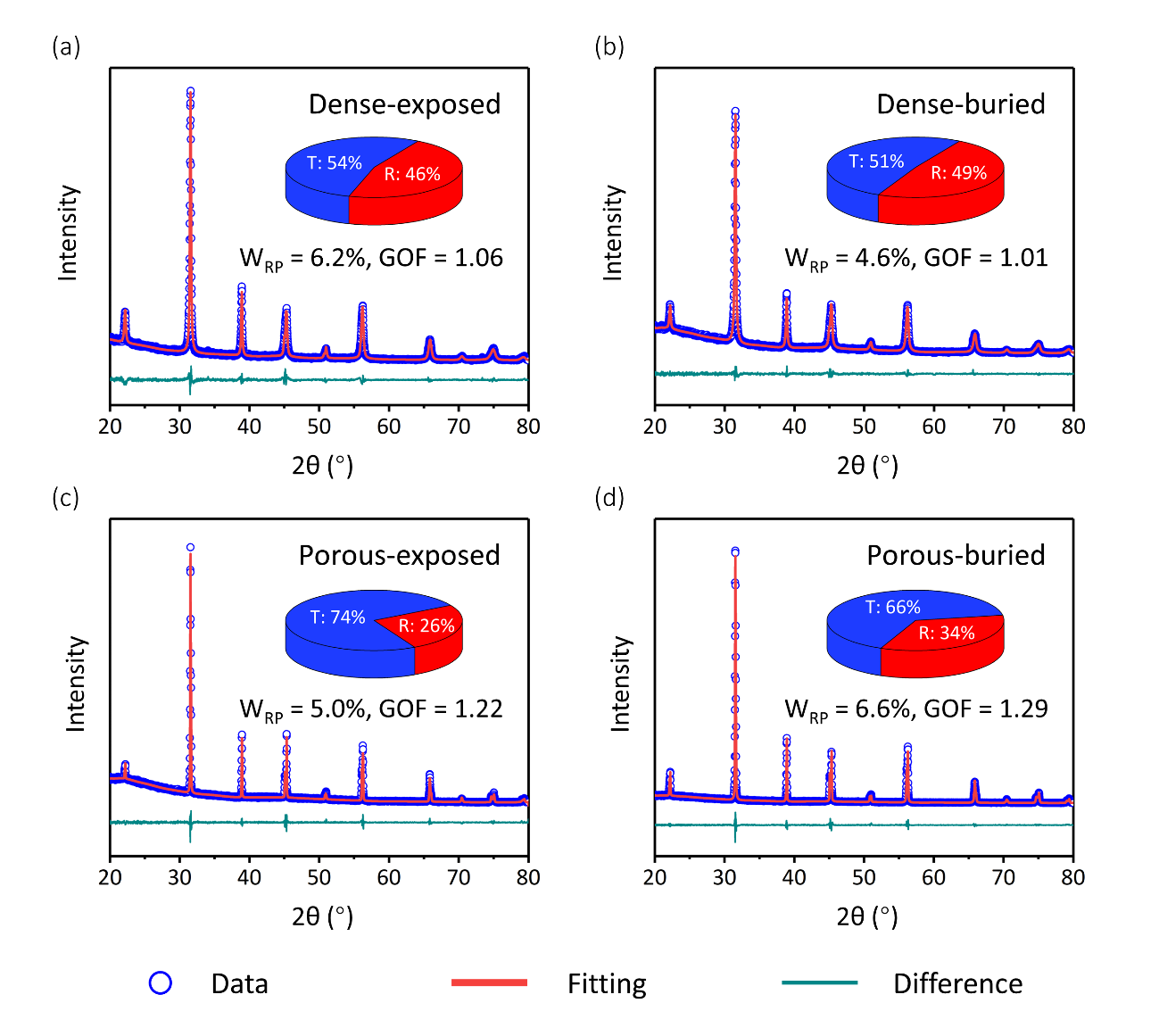
**

**Figure S9** Rietveld refinement of the XRD data of the (a) dense-exposed, (b) dense-buried, (c) porous-exposed and (d) porous-buried BCZT ceramics.

**
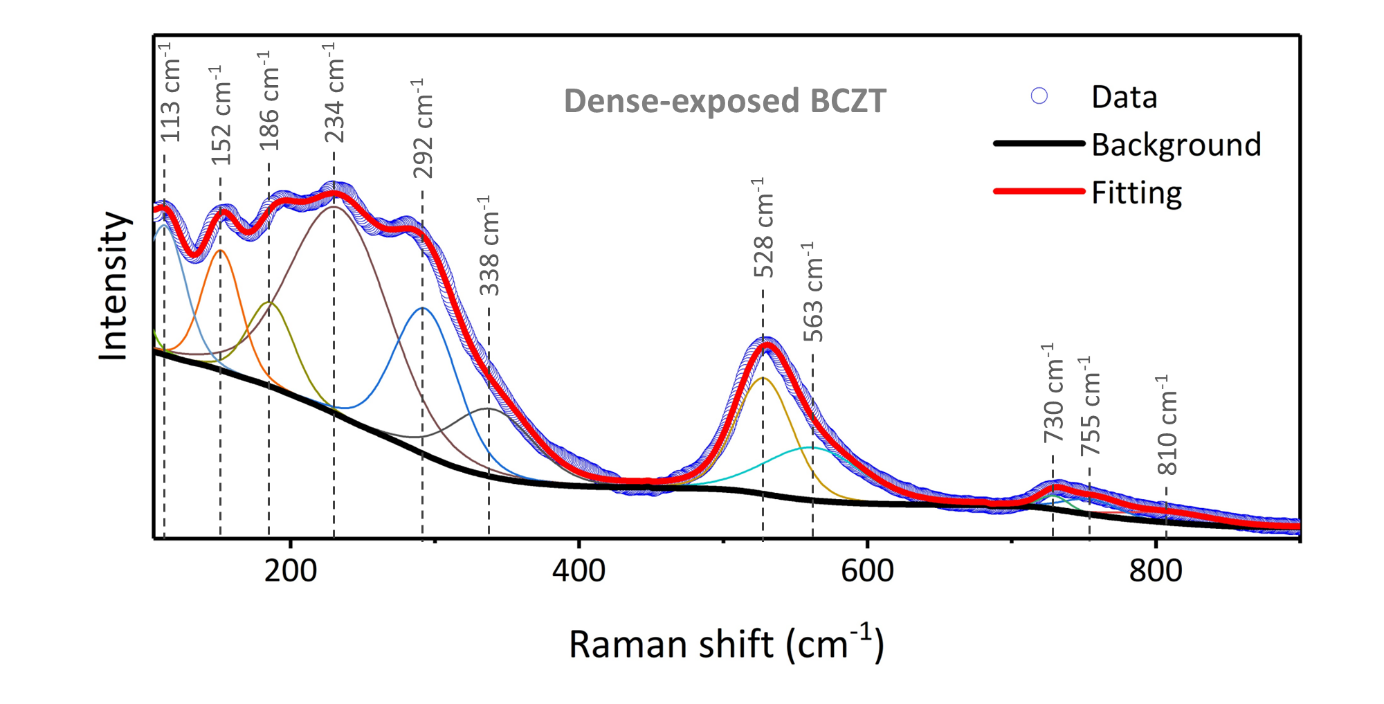
**

**Figure S10** Raman peak assignment of the dense-exposed BCZT.

The Raman vibration modes at

113 cm^-1^: Zr-O motion in the lattice

152 cm^-1^: A_1_(TO_1_)

186 cm^-1^: E(LO_1_), E(TO_2_)

234 cm^-1^: A_1_(TO_2_)

292 cm^-1^: B_1_

338 cm^-1^: E(LO_2_)

528 cm^-1^: A_1_(TO_3_)

563 cm^-1^: phonon mode of barium titanate-based compositions

730 cm^-1^: E(LO_4_), A_1_(LO_3_)

755 cm^-1^: phonon mode, splitting of the LO mode

810 cm^-1^: A_1g_ breathing-like mode due to the presence of two B-site species of Ti and Zr

**
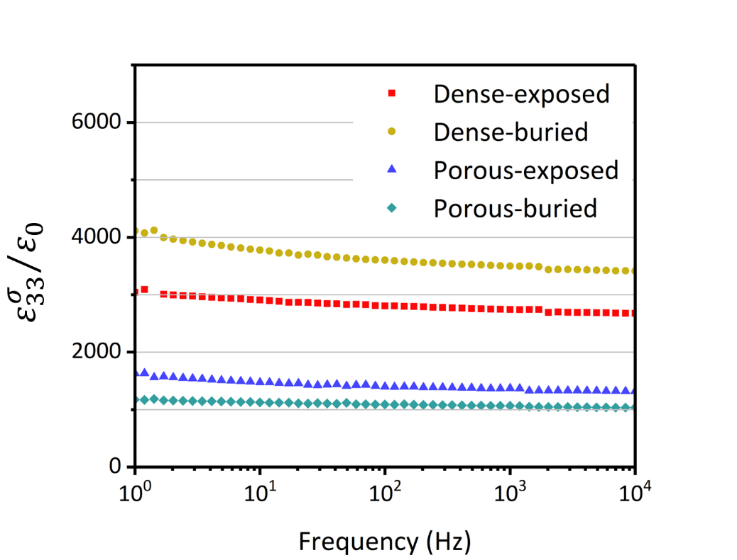
**

**Figure S11** Relative permittivity at constant stress (${\varepsilon_{33}^{\sigma}}/{\varepsilon_{0}}$) of the poled BCZT ceramics.


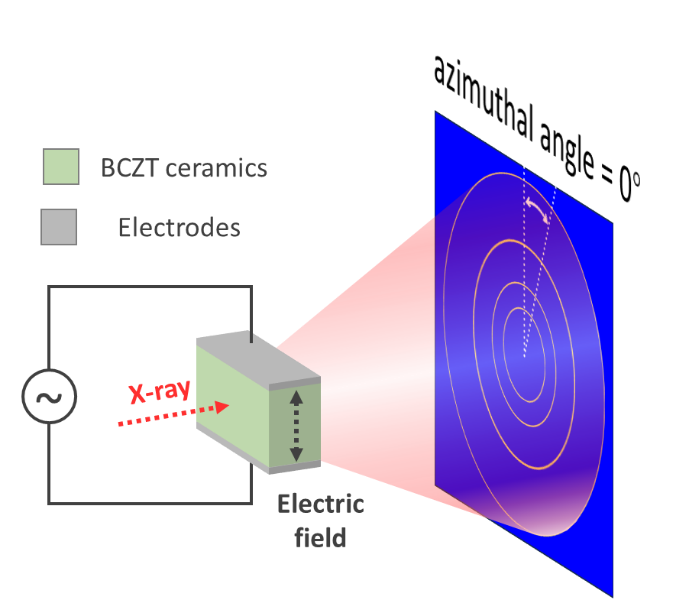


**Figure S12** Schematic of the synchrotron X-Ray diffraction process.

**
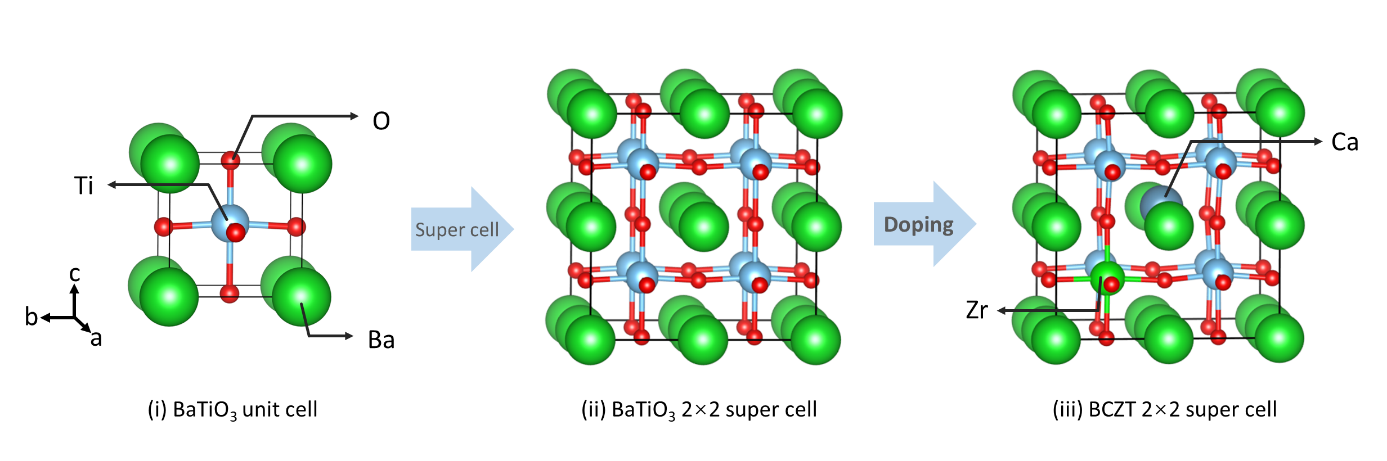
**

**Figure S13** Process flow of DFT calculation of the BCZT lattice structure.


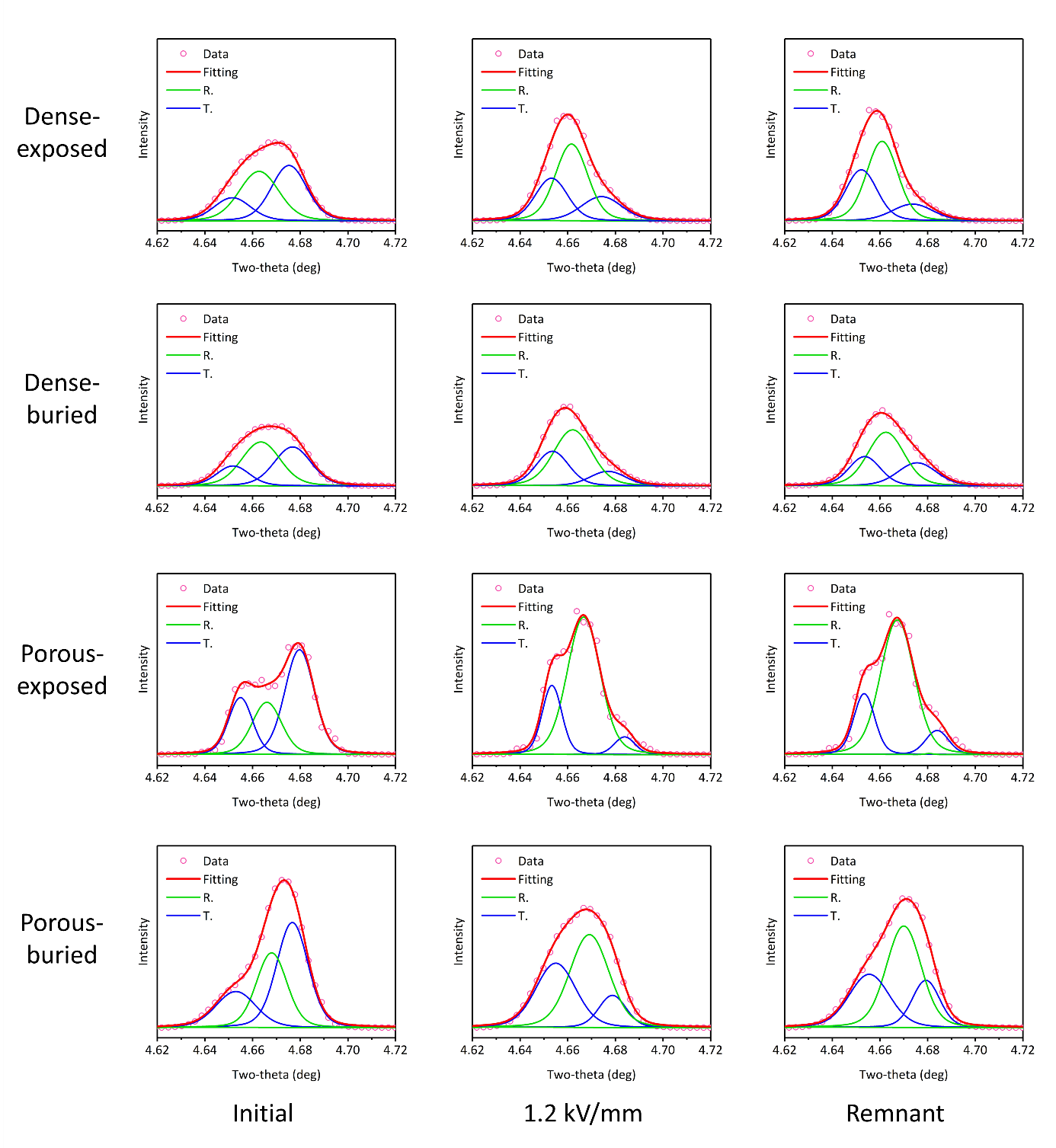


**Figure S14** Peak assignment of the synchrotron X-Ray diffraction of the BCZT ceramics at (200) with in-situ applying alternative electric field, (R. = rhombohedral phase, T. = tetragonal phase).


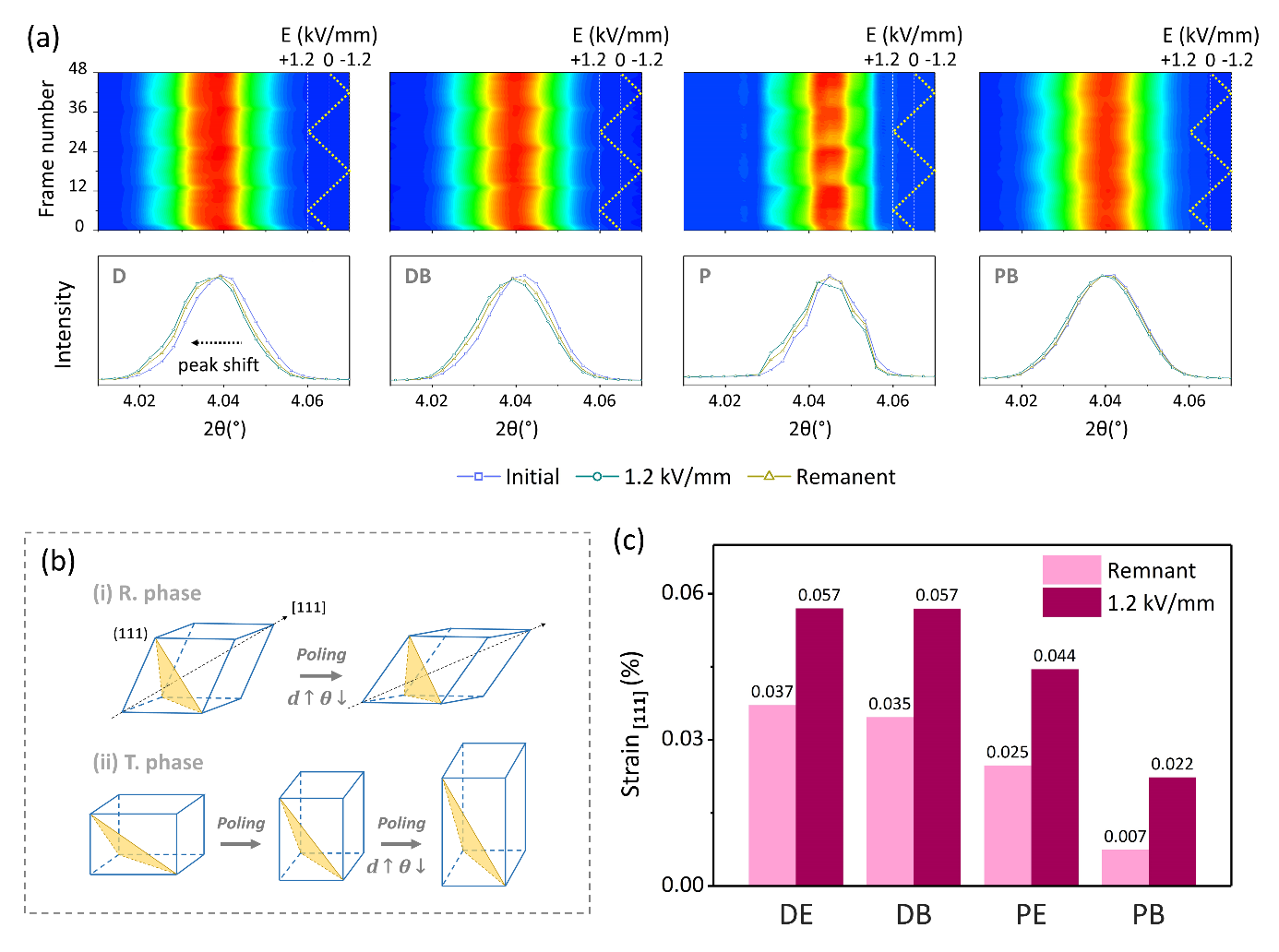


**Figure S15** (a) Synchrotron XRD at (100) with in-situ applying alternating electric field with a magnitude of 1.2 kV/mm. (b) Schematic of how the lattice distance changed, with the lattice distortion induced by the poling field in the (i) rhombohedral (R.) phase and (ii) tetragonal (T.) phase. (c) peak position of the BCZT ceramics. (DE = dense-exposed, DB = dense-buried, PE = porous-exposed, PB = porous-buried).


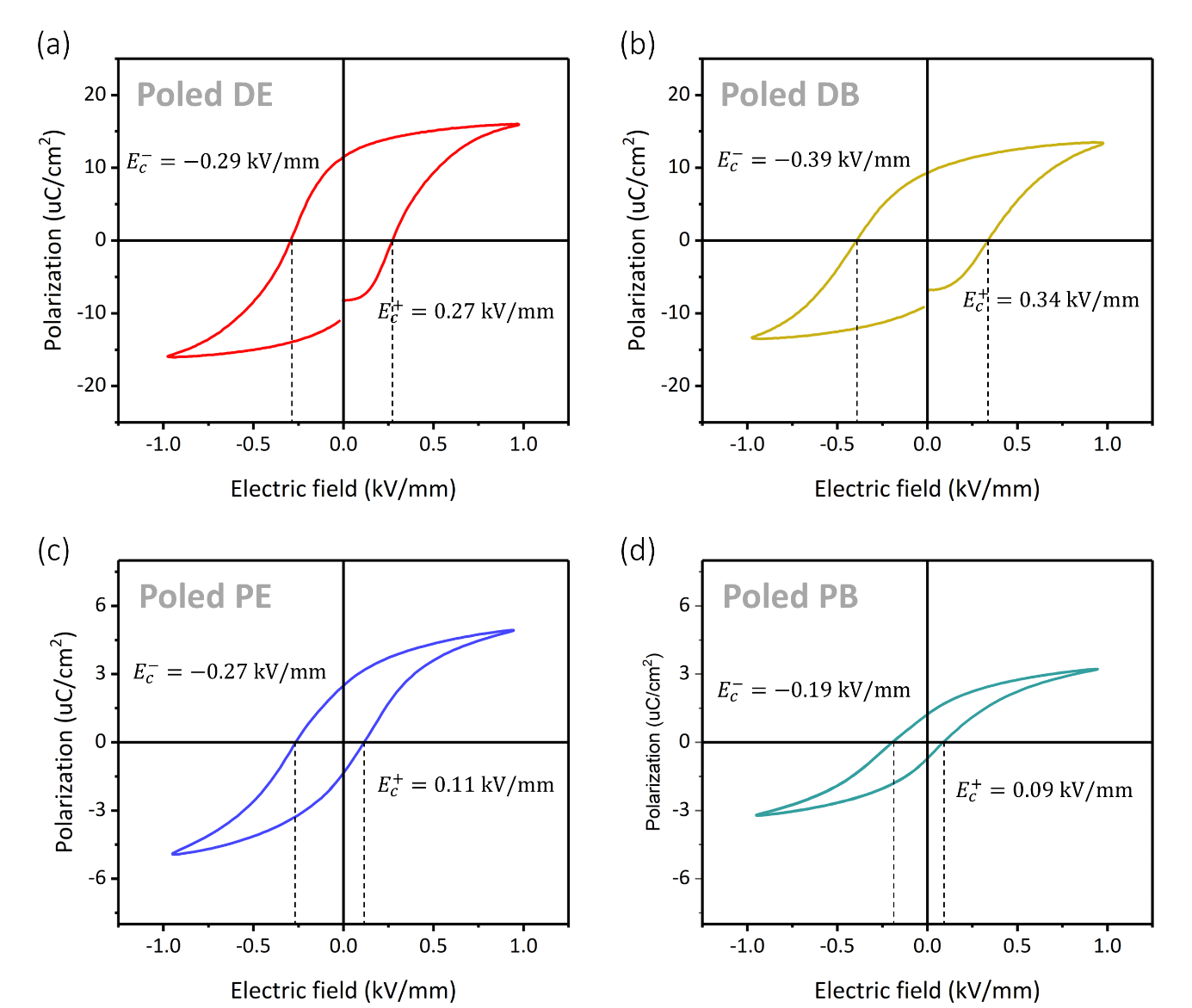


**Figure S16** P-E loop of the poled BCZT ceramics at 100 Hz: (a) dense-exposed (DE), (b) dense-buried (DB), (c) porous-exposed (PE) and (d) porous-buried (PB).

The internal bias field ($E_{bias}$) is calculated from the positive coercive field ($E_{c}^{+}$) and the negative ($E_{c}^{-}$), as below.

$$\begin{aligned} E_{bias}=\frac{E_{c}^{+}+E_{c}^{-}}{2}\#\left( S1 \right) \end{aligned}$$

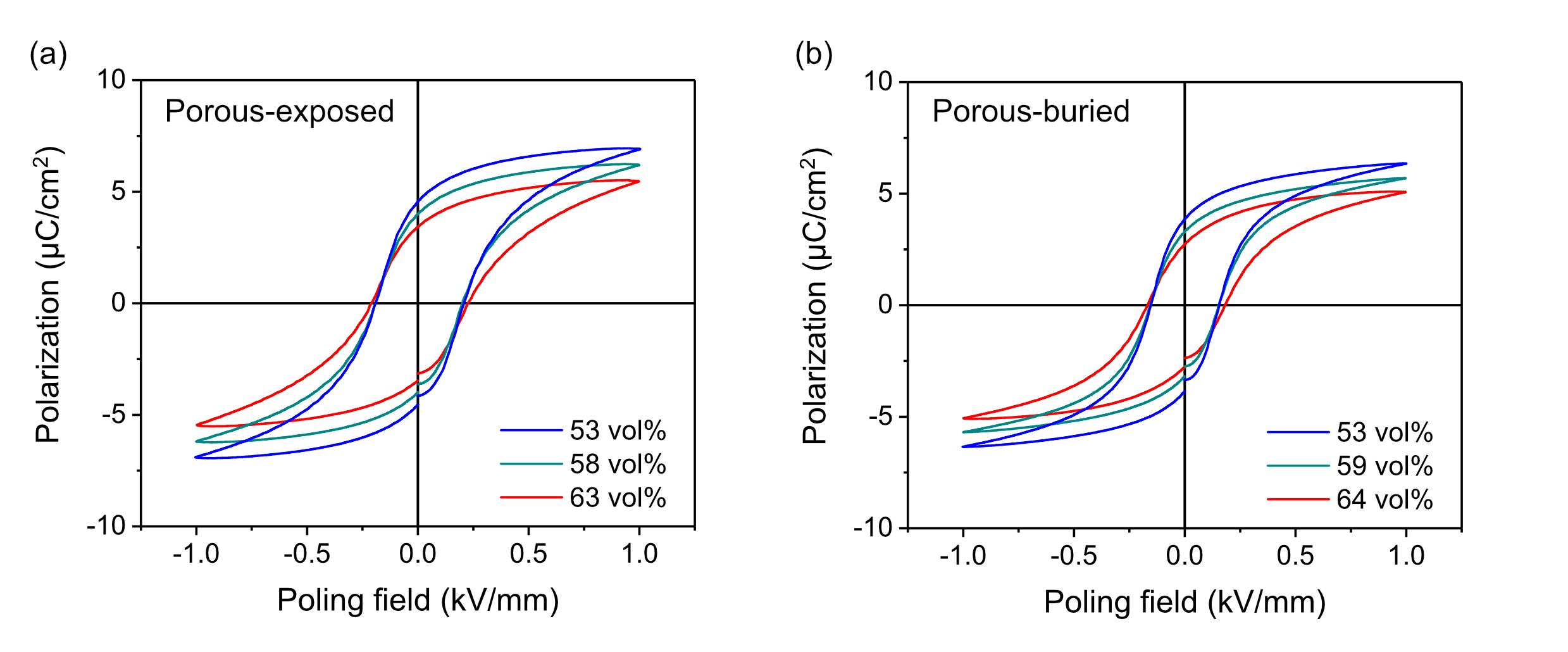


**Figure S17** P-E loop of the porous BCZT with different porosity: (a) porous-exposed BCZT and (b) porous-buried BCZT.

**
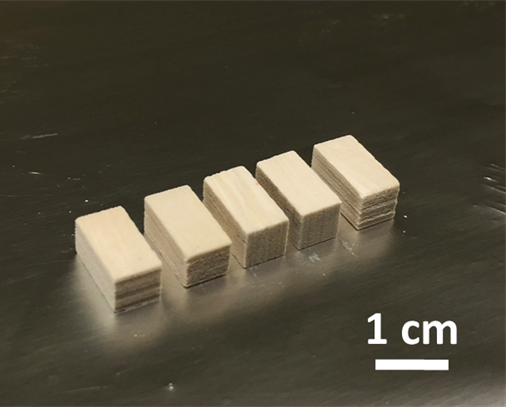
**

**Figure S18** Optical photograph of the BCZT bars processed into dimensions of 5×5×10 mm^3^ for the energy harvesting power output testing.

**Energy flow model of piezoelectric harvesting process**

As shown in **Figure S19**, the energy harvesting model includes three stages: (i) *energy extraction*, where the energy is extracted from the external mechanical excitation to provide an input mechanical power ($P_{in}$); (ii) *energy conversion*, where $P_{in}$ is converted by the direct piezoelectric effect, generating an electrical power ($P_{gen}$); and (iii) *energy transfer*, where the $P_{gen}$ is transferred to an external load, providing the electrical power output ($P_{out}$).


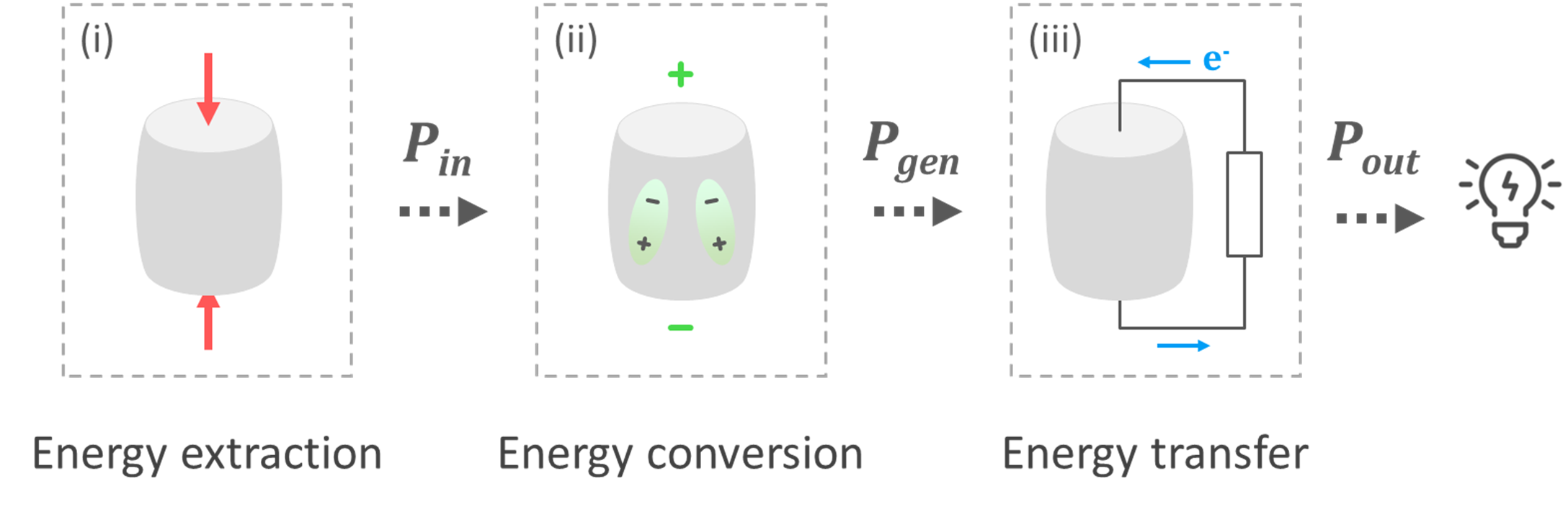


**Figure S19** Schematic of energy flow model of piezoelectric energy harvesting.

In stage one (*energy extraction)*, the input mechanical is dependent on the mechanical impedance ($Z_{m}$) of the harvester, as illustrated in the electrical-mechanical equivalent circuit diagram (**Figure S20**):

$$\begin{aligned} Z_{m}=r+i(\omega m-\frac{c}{\omega})\#\left( S2 \right) \end{aligned}$$

where $r$, $m$ and $c$ are the damping force coefficient, mass and compression stiffness of the harvester. The $c$ was determined with the Young’s modulus of the material, see **Figure S21**, and $\omega$ is the angular speed of the external excitation force. The power of the input mechanical energy stored in the material ($P_{in}$) can then be expressed as below;

$\begin{aligned} P_{in}=\frac{cF_{0}^{2}}{2\pi^{2}f{|Z_{m}|}^{2}}\#\left( S3 \right) \end{aligned}$

where $F_{0}$ is the amplitude of the external excitation force. In the second stage (*energy conversion)*, the $P_{in}$ is converted into electrical power via the piezoelectric effect.

$$\begin{aligned} P_{gen}=k_{33}^{2}P_{in}\#\left( S4 \right) \end{aligned}$$

where $P_{gen}$ is the electrical power generated by the piezoelectric material and $k_{33}^{2}$ is the electromechanical coupling coefficient. To analyze stage three (*energy transfer)*, an equivalent circuit is needed to establish electrical impedance network, see Figure S14. The piezoelectric material is considered a serial connection of a voltage source ($V_{eq}$), an inductor due to the $m$ of the material ($L_{m}$), a resistor due to the $r$ of the material ($R_{r}$) and a capacitor due to the compression stiffness of the material ($C_{c}$), as below

$$\begin{aligned} V_{eq}=\frac{F}{\alpha}\#\left( S5 \right) \end{aligned}$$

$$\begin{aligned} L_{m}=\frac{m}{\alpha^{2}}\#\left( S6 \right) \end{aligned}$$

$$\begin{aligned} R_{r}=\frac{r}{\alpha^{2}}\#\left( S7 \right) \end{aligned}$$

$$\begin{aligned} C_{c}=\frac{\alpha^{2}}{c}\#\left( S8 \right) \end{aligned}$$

where $\alpha$ is the force-voltage factor as

$$\begin{aligned} \alpha=cd_{33}\#\left( S9 \right) \end{aligned}$$

Then, the whole equivalent circuit can be considered a serial connection of the voltage source, the mechanical component ($L_{m}$, $R_{r}$ and $C_{c}$ in series) and the electrical component (the capacitance of the piezoelectric material ($C_{piezo}$), a load resistance ($R_{load}$) and the electrometer ($Z_{elect}$) in parallel). The electrical impedance of the mechanical component is;

$$\begin{aligned} Z_{mec}=R_{r}+X_{c}+X_{m}\#\left( S10 \right) \end{aligned}$$

The electrical impedance of the electrical component is;

$$\begin{aligned} Z_{elect}=\frac{X_{piezo}R_{load}R_{elect}}{X_{piezo}R_{load}+X_{piezo}R_{elect}+R_{load}R_{elect}}\#\left( S11 \right) \end{aligned}$$

With Eq. (10, 11), the correlation between $V_{eq}$, the open-circuit voltage ($V_{oc}$) and the output voltage ($V_{out}$) can be obtained:

$$\begin{aligned} \frac{V_{out}}{V_{eq}}=\frac{\left| Z_{ele} \right|}{\left| Z_{ele}+Z_{mec} \right|}\#\left( S12 \right) \end{aligned}$$

$$\begin{aligned} \frac{V_{oc}}{V_{eq}}=\frac{\left| X_{piezo} \right|}{\left| X_{piezo}+Z_{mec} \right|}\#\left( S13 \right) \end{aligned}$$

$$\begin{aligned} \frac{V_{out}}{V_{oc}}=\frac{\left| Z_{ele} \right|\left| X_{piezo}+Z_{mec} \right|}{\left| X_{piezo} \right|\left| Z_{ele}+Z_{mec} \right|}\#\left( S14 \right) \end{aligned}$$

Then, the transfer efficiency of the $P_{gen}$ to the output electrical power ($P_{out}$) can be expressed by the $\frac{V_{out}}{V_{oc}}$ as;

$$\begin{aligned} \frac{P_{out}}{P_{gen}}=\frac{1}{2{\omega C_{piezo}R}_{load}}\times\frac{V_{out}^{2}}{{V_{oc}}^{2}}\#\left( S15 \right) \end{aligned}$$

**Figure S22** shows the energy flow of the whole harvesting process of the dense-exposed BCZT and porous-exposed BCZT at the condition of matched electrical impedance ($R_{load}$ = 6 and 12 MΩ, respectively). According to the obtained energy flow result, the significant improvement in the $P_{out}$ of the porous-exposed BCZT was due to the three-fold higher $P_{in}$ in the energy extraction process due to the lower compressive stiffness of the material, the 16% higher conversion efficiency in the energy conversion process due to the higher $k_{33}^{2}$ of the porous material, and a similar transfer efficiency (33% and 34%) for the energy transfer process using matched electrical impedance for both the dense and porous samples.


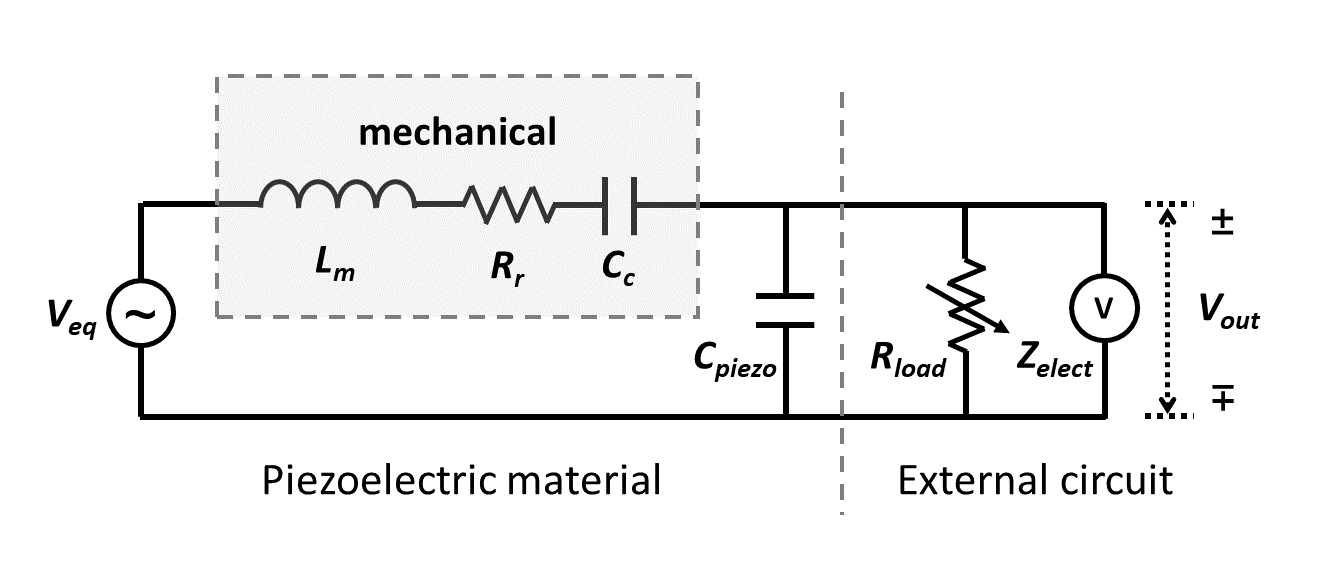


**Figure S20** Equivalent circuit of the piezoelectric harvester for energy flow analysis.


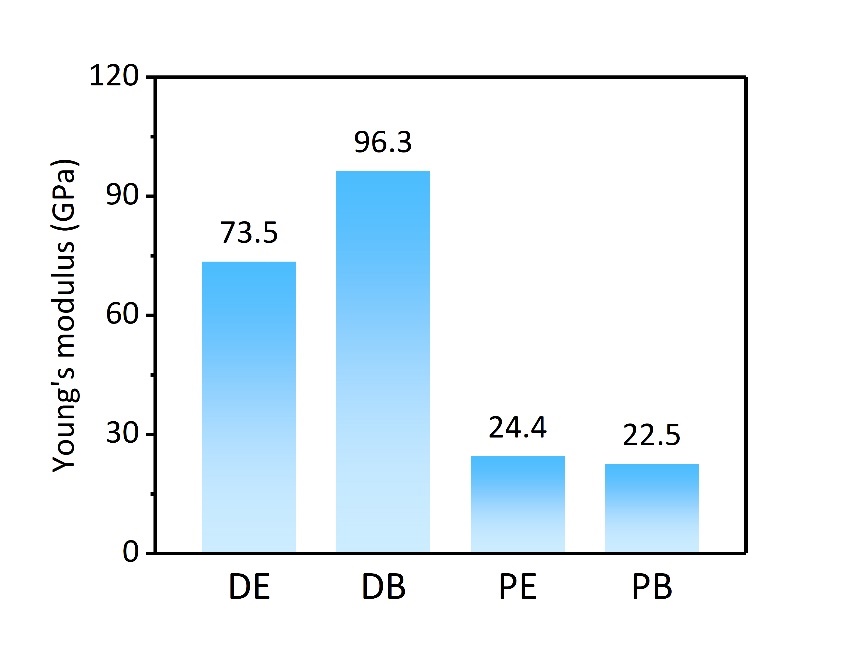


**Figure S21** Young’s modulus of BCZT ceramics calculated with the electromechanical coupling coefficient (DE = dense-exposed, DB = dense-buried, PE = porous-exposed, PB = porous-buried).


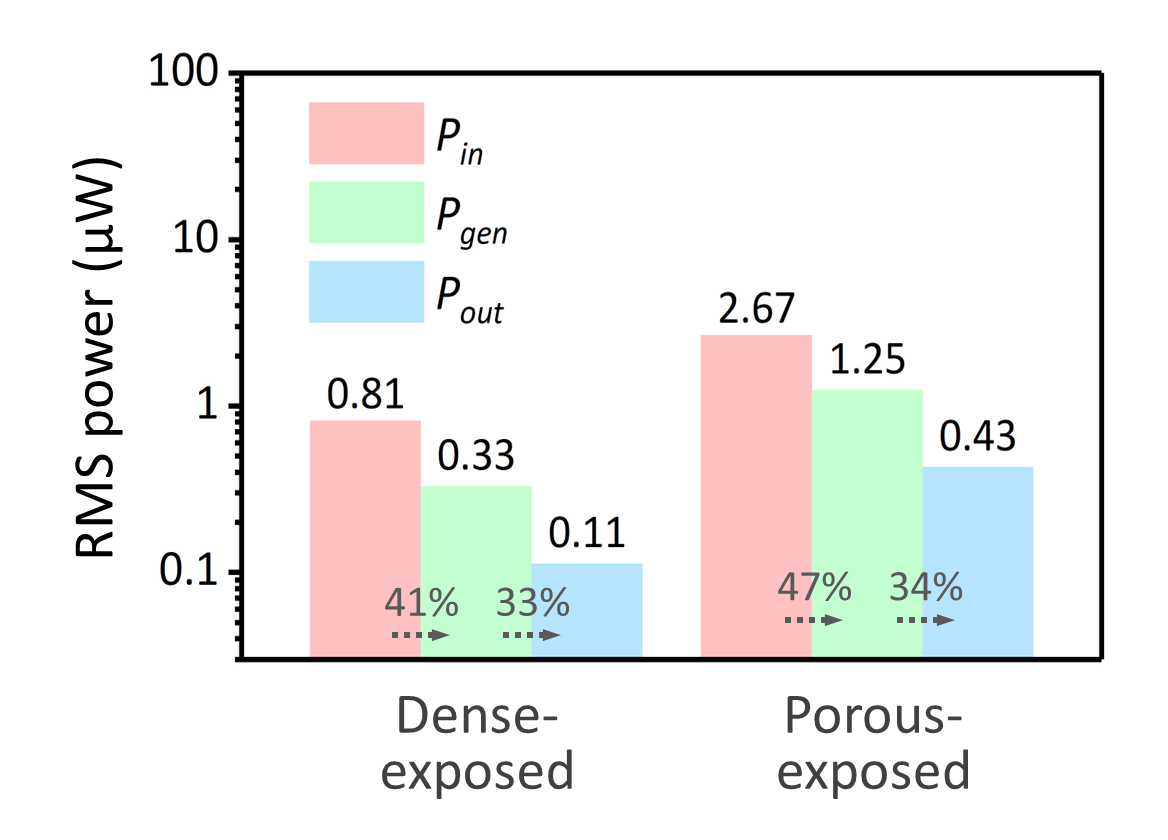


**Figure S22** Energy flow model analysis of the piezoelectric energy harvesting of the dense-exposed and porous-exposed BCZT ceramics fabricated in this work.

**Table S1.** Lattice parameters of the dense, dense buried, porous and porous buried BCZT obtained by XRD refinement (T = tetragonal, R = rhombohedral).

| Samples | Phase | $a$ (Å) | $b$ (Å) | $c$ (Å) | Unit cell volume (Å^3^) | wt.% |
| --- | --- | --- | --- | --- | --- | --- |
| Dense-exposed | T | 3.9955 | - | 4.0255 | 64.26 | 53.8 |
|  | R | 4.0053 | - | - | 64.25 | 46.3 |
| Dense-buried | T | 3.9934 | - | 4.0253 | 64.19 | 48.6 |
|  | R | 4.0042 | - | - | 64.20 | 51.4 |
| Porous-exposed | T | 3.9968 | - | 4.0181 | 64.19 | 74.3 |
|  | R | 4.0072 | - | - | 64.34 | 25.7 |
| Porous-buried | T | 3.9967 | - | 4.0186 | 64.19 | 65.8 |
|  | R | 4.0075 | - | - | 64.36 | 34.2 |

**Table S2.** Data extraction of the polarization-electric field (*P*-*E*) loop of BCZT ceramics with an applied electric field of 1 kV/mm (*P_s_* = saturated polarization, *P_r_* = remnant polarization, *E_c_* = coercive field, *E_bia_* = internal bias field).

| Samples | *P_s_* (μC/cm^2^) | *P_r_* (μC/cm^2^) | *P_r_* / *P_s_* | *E_c_* (kV/mm) | *E_bia_* (kV/mm) |
| --- | --- | --- | --- | --- | --- |
| Dense-exposed | 17.5 | 12.4 | 71% | 0.21 | 0.002 |
| Dense-buried | 16.3 | 11.0 | 67% | 0.26 | 0.002 |
| Porous-exposed with 58.2 vol% porosity | 6.2 | 4.0 | 65% | 0.19 | 0.003 |
| Porous-buried with 58.8 vol% porosity | 5.7 | 3.2 | 56% | 0.15 | 0.001 |
| Freeze-casting BCZT with 49 vol% porosity in Ref. ^[1]^ | 2 | 0.75 | 38% | 0.15 | - |
| Dense BCZT in Ref. ^[2]^ | 18.8 | 10.2 | 54% | 0.15 | - |

**Table S3.** Piezoelectric properties of the BCZT ceramics including longitudinal piezoelectric charge coefficient ($d_{33}$), relatively permittivity (${\varepsilon_{33}^{\sigma}}/{\varepsilon_{0}}$) at 100 Hz, longitudinal voltage output constant ($g_{33}$) at 100 Hz, longitudinal piezoelectric energy harvesting figure of merit (${FoM}_{33}$) at 100 Hz, electromechanical coupling coefficient of the thickness mode ($k_{33}^{2}$), dielectric loss ($\tan\delta$) at 10 kHz and mechanical quality factor ($Q_{m}$).

| Samples | $d_{33}$ (pC/N) | ${\varepsilon_{33}^{\sigma}}/{\varepsilon_{0}}$ (at 100 Hz) | $g_{33}$ (10^-3^ V·m/N) | ${FoM}_{33}$ (10^-12^ m^2^/N) | $k_{33}^{2}$ (%) | $\tan\delta$ (%) | $Q_{m}$ |
| --- | --- | --- | --- | --- | --- | --- | --- |
| Dense-exposed | 372 ± 4.5 | 3000 | 14.0 | 5.21 | 40.5 | 1.55 | 163 |
| Dense-buried | 336 ± 13.2 | 3614 | 10.5 | 3.54 | 35.1 | 1.54 | 213 |
| Porous-exposed with 58.2 vol% porosity | 488 ± 19.2 | 1390 | 39.7 | 19.35 | 46.7 | 2.20 | 89 |
| Porous-buried with 58.8 vol% porosity | 338 ± 8.7 | 1093 | 34.9 | 11.81 | 26.6 | 1.64 | 121 |
| Freeze-casting BCZT with 49 vol% porosity in Ref. ^[1]^ | 410 | 1266 | 35 | 15 | - | - | - |
| Dense BCZT in Ref. ^[2]^ | 540 | 4338 | 14 | 7.6 | 52 | 1.8 | - |

In this work, although the introduction of aligned porosity into BCZT has been shown to increase the $d_{33}$ compared to its dense counterpart, the piezoelectric properties of the porous-exposed BCZT were still at the similar magnitudes to freeze-cast BCZT with a similar porosity level fabricated in other work. This may be because the $d_{33}$ of the dense-exposed BCZT fabricated in this work, 372 pC/N, was relatively low compared to the dense BCZT fabricated in other works with $d_{33}$ of ~500 pC/N ^[2]^. Nevertheless, the increased $d_{33}$ of the exposed-porous BCZT in this work is higher than other porous materials in the literature.

**Table S4.** DFT lattice parameter and spontaneous polarization of the 2×2 supercell BCZT in different crystal phases.

| Crystal structures | Lattice parameters | | Unit cell volume (Å^3^) | Spontaneous polarization ($P_{0}$) |
| --- | --- | --- | --- | --- |
|  | $a$ (Å) | $c$ (Å) |  |  |
| Cubic | 4.0401 | - | 65.944 | - |
| Tetragonal | 4.0319 | 4.0773 | 66.281 | 38.6 μC/cm^2^ in (001) |
| Rhombohedral | 4.0427 | - | 66.072 | 3.4 μC/cm^2^ in (111) |

**References**

[1] W. Sun, J. Zhang, Z. Shi, L. Li, Y. Zhang, Y. Gao, S. Li, *J Eur Ceram Soc* **2024**, *44*, 7605.

[2] X. Wang, Y. Huan, Y. Zhu, P. Zhang, W. Yang, P. Li, T. Wei, L. Li, X. Wang, *Journal of Advanced Ceramics* **2022**, *11*, 184.
